# Seeds as Potential Sources of Phenolic Compounds and Minerals for the Indian Population

**DOI:** 10.3390/molecules27103184

**Published:** 2022-05-17

**Authors:** Pravin Kumar Sahu, Ana Cervera-Mata, Suryakant Chakradhari, Khageshwar Singh Patel, Erick K. Towett, José J. Quesada-Granados, Pablo Martín-Ramos, José A. Rufián-Henares

**Affiliations:** 1 School of Studies Environmental Science, Pt. Ravishankar Shukla University, Raipur 492010, India; sahu.pravin89@gmail.com (P.K.S.); suryachakradhari99@gmail.com (S.C.); 2 Departamento de Edafología y Química Agrícola, Facultad de Farmacia, Universidad de Granada, 18071 Granada, Spain; anacervera@ugr.es; 3 School of Agronomy, Amity University, Baloda-Bazar Road, Raipur 493225, India; kspatel@rpr.amity.edu; 4 World Agroforestry Centre, P.O. Box 30677, Nairobi 00100, Kenya; etowett@gmail.com; 5 Departamento de Nutrición y Bromatología, Instituto de Nutrición y Tecnología de Alimentos, Centro de Investigación Biomédica, Universidad de Granada, 18071 Granada, Spain; quesadag@ugr.es; 6 Department of Agricultural and Environmental Sciences, EPS, Instituto de Investigación en Ciencias Ambientales de Aragón (IUCA), University of Zaragoza, Carretera de Cuarte, s/n, 22071 Huesca, Spain; pmr@unizar.es; 7 Institute de Investigación Biosanitaria ibs. Granada, Universidad de Granada, 18071 Granada, Spain

**Keywords:** seeds, polyphenols, flavonoids, mineral elements

## Abstract

Seeds are major sources of nutrients and bioactive compounds for human beings. In this work, the chemical composition and physicochemical properties of 155 Indian seeds (belonging to 49 families) are reported. Moisture and ash were measured with reference protocols from AOAC; total polyphenols and flavonoids were measured with spectrophotometric methods after extraction with organic solvents, and mineral elements were determined by X-ray fluorescence spectrophotometry. Total phenolic compounds, flavonoids and mineral contents (Al, Ba, Ca, Cl, Co, Cr, Cu, Fe, K, Mg, Mn, Mo, Na, P, Rb, S, Sr, Ti, V and Zn) were found to vary in the ranges 182–5000, 110–4465 and 687–7904 mg/100 g (DW), respectively. Noticeably, polyphenol contents higher than 2750 mg/100 g were observed in 18 seeds. In addition, mineral contents >5000 mg/100 g were detected in the seeds from *Cuminum cyminum*, *Foeniculum vulgare*, *Commiphora wightii*, *Parkia javanica*, *Putranjiva roxburghii*, *Santalum album* and *Strychnos potatorum*. Botanical and taxonomical variations in the proximate characteristics of the examined seeds are also discussed.

## 1. Introduction

In the last decade, a growing interest in seeds as significant ingredients of the daily diet has been observed, since seeds are placed next to legumes as a source of plant proteins [1]. In addition, seeds contribute to meeting the increasing food demand, and in many cases, are also used as traditional medicines [2,3]. Even more, their seed cakes are used for animal feed and as green manures in organic agriculture [4,5].

Seeds are dried products with low water content [1]. Owing to their evolutionary adaptation to the embryonic nutrition of the plants they originate from, seeds are rich in different nutrients, such as proteins, carbohydrates and lipids [6]. In addition, seeds are also good sources of different bioactive compounds, such as carotenoids (vitamin A), tocopherols (vitamin E), xanthophylls and polyphenols [7,8]. Indeed, phenolic compounds such as phenolic acids, flavonoids, stilbenes and lignans are strong antioxidant compounds [9,10]. Polyphenols compounds are the subject of increasing scientific interest due to their potential applicability in the treatment of some chronic diseases, such as cardiovascular diseases, diabetes, osteoporosis or neurodegenerative disorders [11]. Moreover, phenolic compounds can be found in the coats (hull, husk or skin, for instance) covering the cotyledon(s) of seeds [12] and can be even separated from the seed matrix by extraction with an appropriate solvent [13].

Although the phenolic contents of some common seeds have been reported in the literature [14,15,16], less attention has been paid to their potential absorption [17], which affects their potential health effects [18]. For the analysis of total phenols in any kind of vegetables matrixes, the Folin–Ciocalteu method is widely accepted. This is a spectrophotometric method that measures oxidized phenolic compounds using the Folin–Ciocalteu reagent at 750 nm [19]. On the contrary, single phenolic species (such as flavonoids or phenolic acids) are also analyzed by high-performance liquid chromatography coupled with mass spectrometry [20] or diode-array detectors [21].

On the other hand, seeds are important dietary sources of minerals since they accumulate these compounds during plant growth to be used for further development needs [22]. The content of nutrients varies depending on plant variety, agricultural practices, soil and climatic conditions, as well as technological and culinary practices [1]. Mineral elements such as Ca, Co, Cr, Cu, Fe, K, Mg, Mn, Mo, Na, P, S, Se, Zn, etc. have a relevant role in human health [23]. For example, seeds are low in Na and rich in K, and clinical trials and metanalyses suggest that a high intake of potassium is linked with blood pressure reduction [24]. In the case of seeds, the mineral contents of usual seeds are known [25,26], but, for instance, the potential of seeds from herbal plants has received less attention from the scientific community.

When seeds are harvested, their quality characteristics usually decay due to the poor techniques followed by the Indian population (improper sun drying or storage in gunny bags) [27]. In order to limit the effect of the postharvest loss, preservation techniques should be standardized with cheap and easy techniques acceptable by local tribal people as well as manufacturers. Among them, drying is a traditional and effective method used for seeds preservation. In this sense, sun drying combined with hot-air drying are cheap and easily applicable to different seeds [28].

Hence, the aim of this work is to study the phenolic and mineral contents of seeds from 155 species (57 herbs, 14 shrubs, 20 vines and 64 trees) with nutritional interest, belonging to 49 families. The proximate characteristics (visual seed color, seed mass, seed coat mass, moisture and ash contents) are also discussed.

## 2. Results and Discussion

### 2.1. Physicochemical Characteristics

The seeds from the species under study are shown in Figure S1, and their physicochemical characteristics are summarized in Table 1. Their mass ranged from 0.21 to 23,623 mg/seed, with the maximum value having been found for the seeds from *Anthocephalus indicus* (syn. Breonia chinensis). These values are in line with those reported by Cervera-Mata et al. [29], although in that paper, the highest masses were obtained for *Cicer arietinum*, *Phaseolus vulgaris*, *Arachis hypogaea* and *Caesalpina crista*. The mean mass values of the seeds from herbs (*n* = 57), shrubs (*n* = 14), vines (*n* = 20) and trees (*n* = 64) were 125, 556, 1264 and 1629 mg/seed, respectively. Among them, the seeds from *Anthocephalus indicus*, *Anacardium occidentale*, *Areca catechu*, *Artocarpus heterophyllus*, *Bauhinia vahlii*, *Butea frondosa*, *Diospyros melanoxylon*, *Ficus racemosa*, *Gardenia thunbergia*, *Juglans regia*, *Litchi chinesis*, *Lagerstroemia parviflora*, *Mangifera indica*, *Nelumbo nucifera*, *Pistacia vera*, *Phoenix dactylifera*, *Pongamia pinnata*, *Prunus dulcis*, *Sapindus emarginatus*, *Semecarpus anacardium*, *Sterculia foetida*, *Syzygium cumini* and *Trapa natans* (mostly trees) featured the largest seed sizes (>1000 mg/seed). Out of the 155 species studied, 81 had measurable seed coats, whose fractions varied from 4.0% to 95%, with the highest value having been found for *Phyllanthus emblica*. Such values are larger than those previously reported [29], since the coat fractions analyzed in that paper never exceeded 40%. The moisture and ash contents of the studied seeds were in the 1.0–35.5% and 1.0–7.8% ranges, respectively (Table 1). These results are in line with those of other Indian seeds [29]. In fact, moisture and ash content of *Sesamum indicum* seeds were similar to those reported for sesame seeds from Turkey, Sudan and Nigeria [30].

### 2.2. Polyphenol Contents

The concentrations of total polyphenols and flavonoids in the seeds from the 155 species (Table 1) varied from 182 to 5000 mg/100 g and from 110 to 4465 mg/100 g, respectively, with a Flavonoid/Total phenolic compounds ratio ranging from 0.05 to 0.95, which is similar to the ratio previously described for other Indian seeds [29]. The highest levels of total phenolic compounds (at least twice the mean value, 2784 mg/100 g) were identified in the seeds from *Stevia rebaudiana*, *Bixa orellana*, *Momordica charantia*, *Solena amplexicaulis*, *Shorea robusta*, *Diospyros melanoxylon*, *Caesalpinia pulcherrima*, *Hardwickia binata*, *Pterocarpus marsupium*, *Melia azedarach*, *Syzygium cumini*, *Argemone mexicana*, *Papaver somniferum*, *Passiflora foetida*, *Bridelia retusa*, *Nigella sativa*, *Murraya koenigii* and *Sesamum radiatum*.

For comparison purposes, some of the highest values of total phenols (5460–15,188 mg/100 g) have been reported for clove (*Syzygium aromaticum*), peppermint (*Mentha balsamea*) and star anise (*Illicium verum*), according to Pérez-Jiménez et al. [9]. In turn, total phenolic compounds in the 21.2–417 mg/100 g range have been reported for the germinated peanut, *Coriandrum sativum*, cereals, pulses and other seeds [14,16]. These are important results, since Syed et al. [31] reported that peanuts have a high content of polyphenols, with a positive effect on non-communicable diseases such as cancer [32] or diabetes [33]. In fact, more than 20 bioactive compounds with phenol structure have been reported for this botanical species [34]. The total polyphenols content was also similar for other species, such as *Allium cepa* seeds, reported in the range of 200–400 mg/100 g by other authors [35,36]. In this respect, Žilic et al. [37] demonstrated that polyphenols play an important role in sunflower seed oil, by protecting it from oxidation during storage. Again, our total phenols value for *Sesamum indicum* are in line with those provided in other papers [30,38]. It is known that the total phenolic content can differ among the samples from different countries due to variations in the genotypes, ecological factors and cultivation practices [39].

Regarding total flavonoids content, high concentrations (at least twice the mean value, 1648 mg/100 g) were observed in the seeds from *Semecarpus anacardium*, *Bixa orellana*, *Celastrus paniculatus*, *Shorea robusta*, *Caesalpinia pulcherrima*, *Careya arborea*, *Azadirachta indica*, *Melia azedarach*, *Abelmoschus moschatus*, *Thespesia populnea, Syzygium cumini*, *Argemone mexicana*, *Papaver somniferum*, *Passiflora foetida*, *Sesamum indicum*, *Piper nigrum*, *Bridelia retusa*, *Nigella sativa*, *Murraya koenigii* and *Schleichera oleosa*. These levels are quite similar to those reported for Indian weeds [40]. Gobalakrishnan et al. [41] identified ten bioactive compounds, including flavonoids, in *Ludwigia parviflora* seeds (Onagraceae family), also from India, which makes them candidates for the treatment of various diseases. On the other hand, the total flavonoids levels of our study are in line with those reported by other papers [41,42,43,44]. In fact, flavonoids species such as hyperoside, rutin, hesperidin, vicenin, diosmin, luteolin, apigenin, orientine, dihydroquercetin, catechin and arbutin have been found in *Coriandrum sativum* seeds [45], being bioactive compounds with anthihypertensive, hypocholesterolemic, hypolipidemic, anti-atherogenic and antiarrhythmic effects.

The plant type (herb, vine, shrub and tree) had a remarkable influence on the total phenolic compound seed content, which followed the order vine (1142) < tree (1410) < herb (1432) < shrub (1507 mg/100 g). The flavonoid content showed a slightly different trend, with vine (475) < tree (798) < shrub (884) < herb (960 mg/100 g).

**Figure 1:**
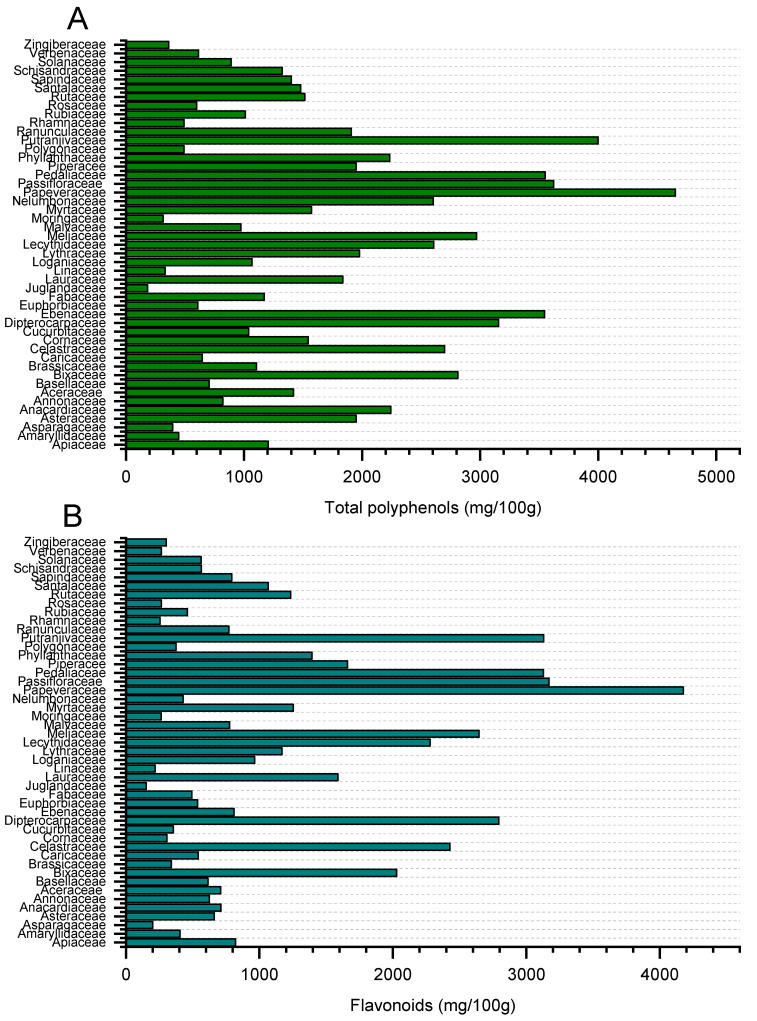
Total polyphenol (panel **A**) and total flavonoid (panel **B**) contents grouped by families.

Total phenols and flavonoids were also grouped by botanical families (Figure 1A,B, respectively). Taxonomically, total phenolic compounds (Figure 1A) followed the sequence: Juglandaceae (182) < Moringaceae (316) < Linaceae (332) < Zingiberaceae (363) < Asparagaceae (395) < Amaryllidaceae (447) < Putranjivaceae (493) < Rosaceae < (598) < Verbenaceae (614) < Moraceae (682) < Basellaceae (705) < Annonaceae (820) < Solanaceae (892) < Malvaceae (974) < Rubiaceae (1011) < Loganiaceae (1068) < Fabaceae (1170) < Apiaceae (1205) < Schisandraceae (1323) < Sapindaceae (1402) < Arecaceae (1420) < Santalaceae (1480) < Rutaceae (1514) < Myrtaceae (1571) < Lauraceae (1836) < Polygonaceae (1908) < Piperacee (1949) ≈ Phyllanthaceae (1949) ≈ Asteraceae (1949) < Lythraceae (1978) < Anacardiaceae(2243) < Nelumbonaceae (2603) < Lecythidaceae (2607) < Lecythidaceae (2607) < Meliaceae(2969) < Phyllanthaceae(3551) < Passifloraceae (3622) < Ranunculaceae (4247) < Papaveraceae (4654 mg/100 g).

Similarly, the flavonoid content in the seeds (Figure 1B) varied from one family to another, with values ranging from 152 to 4178 mg/100 g. The highest contents were detected in four families: Pedaliaceae (3128), Passifloraceae (3170), Ranunculaceae (3750) and Papeveraceae (4178 mg/100 g).

### 2.3. Mineral Contents

Several vegetable species bear seeds during dry season in tropical countries such as India. This time of seed production is very important during periods of food scarcity [46] or for fast-growing populations such as the Indian one [27]. As seeds are excellent sources of micronutrients, their consumption may contribute to meeting the nutritional requirement and to overcoming the micronutrient deficiency at minimum cost [47,48]. Since the largest concentration of people with mineral elements deficiencies lives in low-income South Asian countries, including India [49], and due to the predominantly vegetarian diet of Indian inhabitants [50], a possible solution for this problem could be the increase in seed consumption [51].

**Figure 2:**
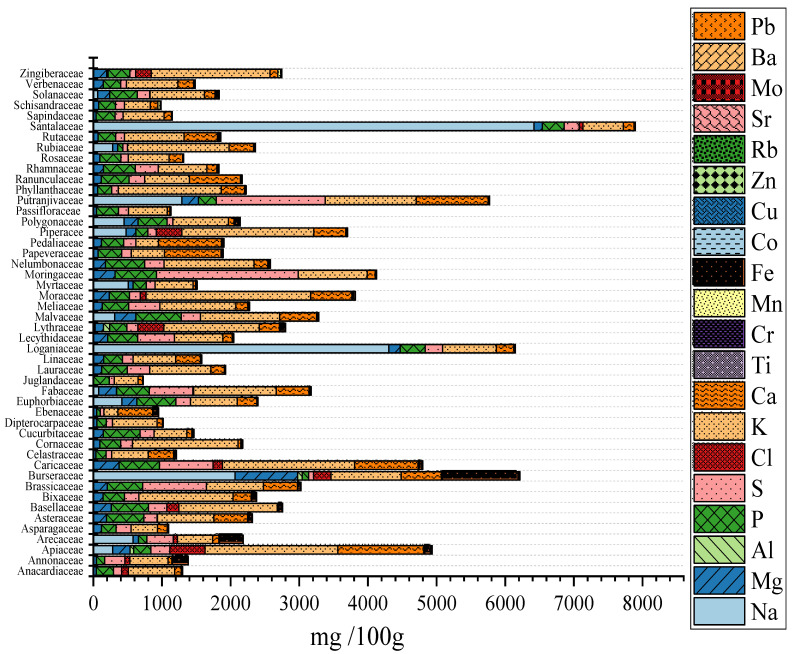
Mineral element content grouped by families.

The contents of 20 mineral elements (Al, Ba, Ca, Cl, Co, Cr, Cu, Fe, K, Mg, Mn, Mo, Na, P, Rb, S, Sr, Ti, V and Zn) in the seeds grown in India from the 155 species under study are shown in Table S1. The mineral content varied from 687 to 7904 mg/100 g (Figure 2). Although these values are generally in line with those previously reported [25,29,52], there are seeds with the largest mineral content exceeding by up to 3000 mg the limit of 4913 mg/100 g found in that paper; this could be related with the lower number of seeds analyzed in that study (60 species vs. 155 species in the current paper) and also related with the botanical nature of seeds. In ref. [29] the studied seeds were mainly beans, weeds and pulses, while in the current paper, a broad variety is found. In this sense, the seed mineral potentiality was categorized in two groups using cluster analysis (data not shown); cluster-I included seeds from 148 plants with mineral contents in the 687–5089 mg/100 g interval, while cluster-II consisted of seven species (*Cuminum cyminum, Foeniculum vulgare, Commiphora wightii, Parkia javanica, Putranjiva roxburghii, Santalum album* and *Strychnos potatorum*), which had mineral contents in the 5728–7904 mg/100 g range.

The mineral accumulation in the seeds followed the following increasing trend: As (0.001) < V (0.01) < Mo (0.04) < Pb (0.05) < Co (0.06) < Cr (0.12) < Ba (0.22) < Ti (0.62 < Cu (1.3) < Rb (1.5) ≈ Sr (1.5) < Zn (4.2) < Al (4.7) < Mn (4.9) < Cl (42) < Fe (43) < Mg (185) < Na (187) < S (354) < Ca (375) < P (408) < K (972 mg/100 g). Nonetheless, it should be taken into consideration that mineral levels are largely influenced by factors such as the soil quality, topology, taxonomy, weather, etc. [39].

In relation to macronutrients, P and S were found to accumulate in all seeds, at concentrations ranging from 53 to 958 mg/100 g and from 50 to 5144 mg/100 g, respectively. These levels are similar to those of other seeds collected in India [29,40]. The highest P and S contents were found in *Corchorus olitorius* and *Parkia javanica* seeds, respectively. In another study [29] *Crotalaria albida*, *Rorippa palustris* and *Cleome viscosa* showed the highest S contents. High P contents (602–661 mg/100 g) and S contents (638–2067 mg/100 g) occurred in seeds belonging to the Moringaceae and Malvaceae families and to the Brassicaceae, Caricaceae, Fabaceae, Moringaceae and Putranjivaceae families, respectively (Figure 2). Phosphorous is an important mineral required for the normal growth and maturity of plants, while S is another essential element for the synthesis of chlorophyll and proteins [53].

Chlorine is required in small amounts for plant metabolism and photosynthesis [53]. However, Cl accumulated in the seeds from 26 species (Table S1) at concentrations ranging from 14 to 1079 mg/100 g. The highest contents were found in Apiaceae seeds, followed by Lythraceae and Piperacee seeds (373–376 mg/100 g). The maximum value was observed in *Trapa natans* seeds, followed by *Cuminum cyminum*. In general, these values are in line with those of *Heliotropium indicum* (300 mg/100 g) [27].

Sodium was detected in the seeds from 17 species (78–6419 mg/100 g), with the highest contents in the seeds from *Santalum album* and *Strychnos potatorum*. In a previous publication [29], high Na accumulation was detected in the seeds from *Eleusine corocana*, *Oryza sativa*, *Setaria italica* and *Zea mays*. In general, some plants tend to accumulate Na when they grow in saline soils, secreting salts to regulate the ion balance, contributing to salinity tolerance [54]. In the case of potassium, it was identified in seeds from all species, at concentrations between 21 and 2625 mg/100 g, which are in line with the values found in weeds, pulses and beans from India [29]. The highest K accumulation (>2.5× mean value, 1944 mg/100 g) was identified in *Coriandrum sativum*, *Cuminum cyminum*, *Foeniculum vulgare*, *Butea frondosa*, *Delonix regia*, *Leucaena leucocephala*, *Lagerstroemia parviflora*, *Artocarpus heterophyllus*, *Ficus racemosa* and *Withania coagulans*. Remarkable K contents (1729–2349 mg/100 g) were detected in the seeds from the Apiaceae, Caricaceae, Moraceae, Piperacee and Zingiberaceae families.

Magnesium is the main component of chlorophyll [53] and was identified in the seeds from 147 species, at concentrations between 34 and 910 mg/100 g, in line with other seeds [29,40]. High Mg contents (>395 mg/100 g) were detected in the seeds from *Abelmoschus esculentus*, *Acacia nilotica*, *Cassia fistula*, *Commiphora wightii*, *Corchorus olitorius*, *Parkia javanica*, *Pterocarpus marsupium* and *Urena lobata*, which are similar to those reported for barley [55] and millet seeds [56]. The highest Mg accumulation was noticed in seeds belonging to the Amaryllidaceae, Burseraceae, Caricaceae and Moringaceae families.

Calcium is also an important mineral for human nutrition that was accumulated at concentrations in the 3.0–1786 mg/100 g range in the seeds from 155 species (Table S1). These are values quite similar to those for weeds such as *Panicum sumatrense*, *Setaria italica*, *Cassia tora*, *Heliotropium indicum*, *Rorippa palustris* and *Ludwigia parviflora* [14N]. Abundant Ca accumulation (919–1253 mg/100 g) was observed in the seeds from four families: Apiaceae, Caricaceae, Pedaliaceae and Putranjivaceae (Figure 2).

Strontium content in 125 cultivars varied from 0.1 to 27.4 mg/100 g. These are higher levels than those described for weeds, pulses and beans from India [29,40]. High Sr content (from 21 to 27 mg/100 g) was accumulated in two Apiaceae family species: *Daucus carota spp. Sativus* and *Foeniculum vulgare*. Rubidium concentration in the seeds under study ranged from 0.2 to 13.4 mg/100 g; such high Rb accumulation was registered in *Lepidium sativum* seeds. These levels are similar to those found in other seeds [40] and within the normal range in foods [57].

Barium accumulated in the seeds from 18 species at milligram levels (1.0–7.9 mg/100 g). The maximum Ba content was detected in *Daucus carota spp. Sativus*. Relatively high contents (2.3–2.4 mg/100 g) were observed in Apiaceae and Rubiaceae seeds (Figure 2). Aluminum accumulated in the seeds from eight species over a wide concentration range: from 41 to 269 m/100 g. Aluminum at high levels (240–269 mg/100 g) was found in the seeds from *Trachyspermum ammi* and *Trapa natans*.

Four micronutrients (Mn, Fe, Cu and Zn) accumulated in all cultivars at concentrations in the 0.5–47, 0.1–1100, 0.3–12.6 and 0.4–21 mg/100 g ranges, respectively. These are values similar to those of other seeds from India [40], Senegal [58] and Uganda [59]. High Mn, Fe, Cu and Zn contents (at least twice the 9.8, 46, 2.8 and 8.4 mg/100 g average contents, respectively) were accumulated in *Acacia auriculiformis*, *Allium cepa*, *Amomum subulatum*, *Citrus limon*, *Delonix regia*, *Illicium verum*, *Lantana camara*, *Malachra capitata*, *Mimosa pudica*, *Sida acuta*, *Trachyspermum ammi* and *Urena bobata*; *Areca catechu*, *Annona squamosa*, *Carthamus oxyacanthus*, *Citrus limon*, *Commiphora wightii*, *Lagerstroemia parviflora*, *Phoenix dactylifera*, *Phoenix sylvestris*, *Solanum melongena* and *Trachyspermum ammi*; *Careya arborea*, *Lagenaria siceraria*, *Malachra capitata*, *Mimosa pudica*, *Pterocarpus marsupium*, *Sesbania sesban*, *Sida acuta* and *Syzygium cumini*; *Acacia nilotica*, *Abelmoschus esculentus*, *Anacardium occidentale*, *Basella rubra*, *Brassica oleracea var. botrytis*, *Commiphora wightii*, *Cucumis melo var. cantalupensis*, *Cucumis sativus*, *Sesbania grandiflora*, *Sesbania sesban* and *Ziziphus mauritiana*, respectively. These high amounts of Mn were also associated with high amounts of Ca (Table S1), since Mg can replace Ca in its functions [60]. The high Zn levels of the above-reported seeds are nutritionally relevant, since Zn deficiencies are prevalent in India [51].

Trace elements (Ti, V, Cr, Co, Mo and Pb) were detected in 35, 7, 27, 73, 2, 11 18 and 51 cultivars (Table S1) in the following ranges: 0.9–14, 0.1–0.3, 0.3–3.3, 0.1–0.6, 0.1–0.2, 0.1–0.2, 0.1–2.7 and 0.1–0.5 mg/100 g. The maximum accumulation of Ti and V, Cr, Co, As, and Pb, Se and Mo was observed in *Trachyspermum ammi*, *Bauhinia vahlii*, *Solanum virginianum*, *Phoenix sylvestris*, *Parkia javanica* and *Sesbania grandiflora*. In the case of Pb, the levels of this toxic element are within the normal range of other seeds from India [29,40].

### 2.4. Variations in Mineral Levels as a Function of Plant Type and Family

The mineral concentration variation as a function of plant type is presented in Table 2. Remarkably, high contents of Na and S, Mg-K-Fe-Mo, Al-Cl-Ca-Mn-Co-Ba, P-Cu-Zn were detected in the tree, shrub, herb and vine species, respectively. The taxonomical seed mineral concentration variations are shown in Figure 2 and Table S2. The highest total mineral contents were found in seeds from the Santalaceae, Burseraceae, Lythraceae, Moringaceae, Apiaceae, Moraceae, Papeveraceae, Solanaceae and Arecaceae families.

The nutrients in the soil solution can interact with each other, affecting their absorption and bioavailability [22]. The P/S (*n* = 155), K/P (*n* = 155) and Ca/Mg (*n* = 143) ratios in the seeds were in the 0.06–4.96, 0.24–21.07 and 0.04–12.61 ranges, respectively. Their minimum and maximum values were observed in *Parkia javanica* and *Persicaria punctate*; in *Trapa natans* and *Gardenia thunbergia*; and in *Luffa aegyptiaca* and *Papaver somniferum* seeds, respectively. These are ratios that have been previously described for other seeds from India [29,40].

### 2.5. Statistical Relationship among Mineral and Phytochemical Contents

**Figure 3:**
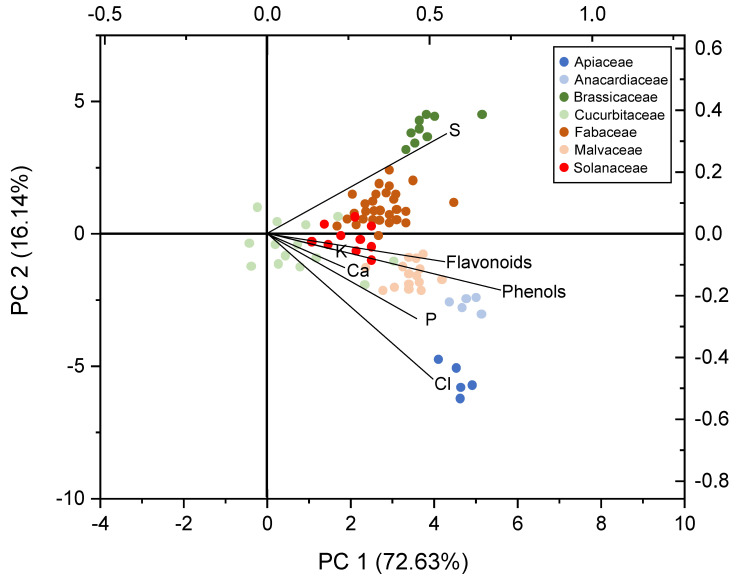
Principal component analysis of mineral and phytochemical contents.

Due to the large number of minerals and polyphenols studied (*n* = 22), a principal component analysis (PCA) was performed to obtain a low number of linear combinations of such parameters that explained data variability. We included those families that were the most representative in the study (with at least five different botanical species). Figure 3 depicts the results obtained, showing the main drivers on the separation of botanical families (total phenols, flavonoids, Ca, Cl, K, P and S). The combination of two components explained 88.77% of the variance. The PCA grouped the seeds from the Apiaceae family due to their high Cl and P contents. On the opposite, samples from the Brassicaceae family grouped in a different sector due to their high S levels. Fabaceae seeds also had an important content of S. Flavonoids and total phenols also played a role, especially for the Anacardiaceae family (Figure 3).

## 3. Materials and Methods

### 3.1. Sample Collection and Preparation

Seeds from 155 species (listed in Table 1), belonging to 49 families, were collected in 2018 in the Raipur area (21.25° N 81.63° E), India, and were identified using standard monographs [52]. For each species, samples from 4 different plants and growing in 4 different locations were collected and were mixed to form composite samples.

The samples were sundried for one week in a glass room and stored in glass bottles. They were further dried for 12 h in a hot-air oven at 50 °C (then, the seeds were stored at −20 °C until analysis). These drying steps could be easily performed by local farmers as well as from small to large companies. The mass was measured using a Mettler–Toledo (Columbus, OH, USA) electronic balance. The testa of 74 examined seeds was carefully removed (manually), and both kernel and seed coat were weighted. Dried seeds or kernels were crushed into powder form using an agate mortar, and particles ≤0.1 mm were sieved out.

Moisture content was determined by drying the samples at 105 ± 2 °C in an air oven for 3 h, and the reported mean values were calculated using the following equation [61]:

% Moisture content = {(w1 − w2)/w1} × 100(1)

where w1 and w2 denote the initial and the dry weight (DW) of the sample, respectively.

Ash residue was evaluated by heating to 550 ± 25 °C in a muffle furnace till constant weight was reached [61] and was reported according to the expression:

% Ash residue = (Wash/Wseed) × 100(2)

where Wash and Wseed denote the weight of the ash residue and the dry weight of the sample, respectively.

### 3.2. Total Phenolic Compound and Flavonoid Determination

The seed samples in powder form (100 mg) were dispersed in 5 mL of an acetone/water (7:3, *v*:*v*) solution and were sonicated in an ultra-sonic bath for 20 min at 20 °C [26]. Then, 5 mL of fresh acetone:water (7:3, *v*:*v*) solution was added to the mixture, and the extraction was repeated for 20 min at 20 °C. After centrifugation, the supernatant was collected [62]. Sigma-Aldrich AR-grade Folin–Ciocalteu reagent, aluminum chloride, tannic acid, gallic acid and quercetin were used for the spectrophotometric determination of phenols. The total phenolic content of each extract was determined as tannic acid equivalents using the Folin–Ciocalteu method [19]. The reaction was carried out in an alkaline medium at room temperature (27 ± 2 °C). After a standing time of 40 min, the absorbance was measured at λ = 725 nm against the reagent blank. A calibration curve was prepared for the absorbance of 2.0, 4.0, 6.0 and 8.0 mg tannin/L. The detection limit (>3 std. dev.) of the method was 0.80 µg/mL as tannic acid. The slope (13.4) and intercept (0) obtained were used for computing the concentration of total phenolic compounds in the sample solution.

The flavonoid content was determined by the aluminum chloride method as quercetin equivalents [63]. Aluminum ions were allowed to react with flavonoids in the presence of tartrate buffer, and the absorbance of the complex formed after a 30 min incubation period was measured at λ = 415 nm against the reagent blank. A calibration curve for 4.0, 6.0, 8.0 and 10.0 mg quercetin/L was prepared. The detection limit of the method (>3 std. dev.) was 0.54 µg/mL as quercetin. The derived slope (20.5) and intercept (0.85) were employed for estimating the concentration of flavonoids in the sample solution.

### 3.3. Mineral Content Determination

A Bruker Tracer i5 spectrometer (Kennewick, WA, USA) was used for the X-ray fluorescence (XRF) analysis of the elements in the samples [64]. Four standard reference materials, brown and white cowpea (*Vigna unguiculata* L. Walp.) seeds, cowpea and mango leaves with reference values were used for validation of the XRF results. The detection limits of the method for the analysis of P, S, Cl, As, Se, Na, K, Rb, Mg, Ca, Sr, Ba, Al, Ti, Cr, Mn, Fe, Co, Cu, Zn, Mo and Pb were 2.4, 3.9, 8.8, 0.1, 0.1, 395, 6.4, 0.1, 79, 3.5, 0.1, 30, 133, 3.5, 1.0, 0.9, 1.4, 1.0, 0.2, 0.2, 0.1 and 0.1 mg/kg, respectively [65,66]. All analyses were carried out in triplicate, and mean values were reported.

### 3.4. Statistical Analysis

Each analysis was carried out in triplicate. A principal component analysis was used to classify the seeds from the 155 species as a function of their mineral and phytochemical contents using Statistica 10.0 (StatSoft, Tulsa, OK, USA) software.

## 4. Conclusions

A wide variety of seeds belonging to herb (57), tree (64), shrub (14) and vine (20) plant types were investigated, focusing on their phenolic and mineral contents. Remarkably, high contents of total phenolic compounds (>4000 mg/100 g) were detected in the seeds from *Argemone mexicana* (Mexican poppy), *Nigella sativa* (black cumin), *Papaver somniferum* (opium poppy), *Sesamum radiatum* (benniseed) and *Solena amplexicaulis* (creeping cucumber). *A. mexicana* and *S. radiatum* also featured the highest flavonoid contents. Regarding mineral levels, *Parkia javanica* and *Santalum album* (Indian sandalwood) featured the highest total concentrations (around 7900 mg/100 g). Taking all this information into account, seeds from different species could be a valuable source of mineral elements and phenolic compounds for the Indian population if such seeds were properly cooked or used as condiments for regular foods.

## Figures and Tables

**Table 1:** Physicochemical characteristics and phenolic compounds of seeds.

No.	Seed	Family	Type	Mass mg	Moisture Content %	Seed Coat %	Ash %	Total Phenols mg/100 g	Flavonoids mg/100 g
1	*Coriandrum sativum* L.	Apiaceae	H	17.87 ± 2.61	7.8 ± 0.2	-	6.8 ± 0.3	481 ± 10	405 ± 9
2	*Cuminum cyminum* L.	Apiaceae	H	3.84 ± 0.09	6.8 ± 0.2	-	5.5 ± 0.1	903 ± 19	815 ± 17
3	*Daucus carota* subsp*. sativus* (Hoffm.) Schübl. & Martens	Apiaceae	H	4.11 ± 0.20	7.5 ± 0.2	-	4.8 ± 0.1	1685 ± 35	1485 ± 31
4	*Foeniculum vulgare* Mill.	Apiaceae	H	4.73 ± 0.38	7.8 ± 0.2	-	4.5 ± 0.1	1056 ± 21	880 ± 18
5	*Trachyspermum ammi* Sprague	Apiaceae	H	1.97 ± 0.09	6.2 ± 0.2	-	6.5 ± 0.2	1899 ± 40	525 ± 11
6	*Allium cepa* L.	Amaryllidaceae	H	3.98 ± 0.19	5.8 ± 0.1	-	5.2 ± 0.1	447 ± 9	405 ± 9
7	*Asparagus racemosus* Willd.	Asparagaceae	H	32.33 ± 2.52	3.2 ± 0.1	-	2.2 ± 0.1	395 ± 8	200 ± 8
8	*Helianthus annuus* L.	Asteraceae	H	50.00 ± 3.12	4.7 ± 0.1	-	4.1 ± 0.1	590 ± 12	490 ± 10
9	*Stevia rebaudiana* Bert.	Asteraceae	H	2.00 ± 0.18	5.5 ± 0.1	-	3.2 ± 0.1	3441 ± 65	256 ± 6
10	*Carthamus oxyacanthus* M.Bieb.	Asteraceae	H	45.35 ± 2.51	4.6 ± 0.1	-	2.8 ± 0.1	1817 ± 38	1240 ± 25
11	*Anacardium occidentale* L.	Anacardiaceae	T	4269 ± 842	2.5 ± 0.1	59 ± 1	1.5 ± 0.1	861 ± 18	122 ± 3
12	*Buchanania lanzan* Spreng.	Anacardiaceae	T	310 ± 57	5.2 ± 0.1	76 ± 2	2.5 ± 0.1	2588 ± 53	406 ± 9
13	*Mangifera indica* L.	Anacardiaceae	T	8336 ± 113	12.9 ± 0.3	46 ± 1	4.5 ± 0.1	2583 ± 52	792 ± 16
14	*Pistacia vera* L.	Anacardiaceae	T	979 ± 100	5.7 ± 0.1	47 ± 1	2.2 ± 0.1	2706 ± 54	190 ± 4
15	*Semecarpus anacardium* L.f.	Anacardiaceae	T	2516 ± 170	1.9 ± 0.1	85 ± 2	2.5 ± 0.1	2476 ± 49	2048 ± 41
16	*Annona squamosa* L.	Annonaceae	T	242 ± 10	6.5 ± 0.2	37 ± 1	3.2 ± 0.1	820 ± 17	626 ± 13
17	*Areca catechu* L. (Indian nut)	Aceraceae	T	4380 ± 557	2.2 ± 0.0	-	1.5 ± 0.0	1553 ± 31	1236 ± 25
18	*Areca catechu* L. (Chikni Supari)	Aceraceae	T	3375 ± 451	2.4 ± 0.1	-	1.4 ± 0.0	2429 ± 50	482 ± 9
19	*Phoenix dactylifera* L.	Aceraceae	T	1065 ± 201	2.1 ± 0.0	-	1.5 ± 0.1	767 ± 16	346 ± 7
20	*Phoenix sylvestris* Roxb.	Aceraceae	T	817 ± 41	1.6 ± 0.0	-	1.8 ± 0.1	930 ± 19	770 ± 15
21	*Basella rubra* L.	Basellaceae	V	38.00 ± 0.20	9.1 ± 0.2	47 ± 1	3.9 ± 0.1	705 ± 14	614 ± 13
22	*Bixa orellana* L.	Bixaceae	S	32.70 ± 2.50	2.5 ± 0.1	-	2.1 ± 0.0	2811 ± 57	2028 ± 42
23	*Brassica campestris* L.	Brassicaceae	H	4.10 ± 0.30	8 ± 0.2	-	4.7 ± 0.8	1471 ± 29	234 ± 5
24	*Brassica hirta* Moench	Brassicaceae	H	4.10 ± 0.30	5.0 ± 0.1	-	4.5 ± 0.1	1902 ± 38	194 ± 4
25	*Brassica nigra* (L.) W.D.J.Koch	Brassicaceae	H	1.02 ± 0.00	7.4 ± 0.1	-	3.2 ± 0.1	1288 ± 26	254 ± 6
26	*Brassica oleracea* var*. capitata* L.	Brassicaceae	H	3.30 ± 0.20	8.6 ± 0.2	-	3.8 ± 0.1	1790 ± 37	242 ± 5
27	*Brassica oleracea* var*. botrytis* L.	Brassicaceae	H	3.20 ± 0.20	8.2 ± 0.3	-	3.7 ± 0.1	972 ± 20	292 ± 6
28	*Brassica rapa* L.	Brassicaceae	H	3.10 ± 0.10	7.3 ± 0.2	-	2.4 ± 0.1	471 ± 18	382 ± 8
29	*Lepidium sativum* L.	Brassicaceae	H	3.10 ± 0.30	6.4 ± 0.1	-	2.1 ± 0.1	378 ± 8	316 ± 7
30	*Raphanus sativus* L.	Brassicaceae	H	10.20 ± 1.60	5.2 ± 0.1	-	3.6 ± 0.1	266 ± 5	238 ± 5
31	*Sisymbrium irio* L.	Brassicaceae	H	0.21 ± 0.10	1.8 ± 0.1	-	2.0 ± 0.1	683 ± 13	590 ± 12
32	*Commiphora wightii* (Arn.) Bhandari	Burseraceae	S	163 ± 18	9.0 ± 0.2	-	4.8 ± 0.1	1834 ± 35	656 ± 13
33	*Carica papaya* L.	Caricaceae	S	15.50 ± 2.20	6.1 ± 0.1	-	5.1 ± 0.1	646 ± 13	542 ± 11
34	*Celastrus paniculatus* Willd.	Celastraceae	V	170 ± 4	2.2 ± 0.1	7.0 ± 0.0	1.5 ± 0.0	2699 ± 54	2428 ± 50
35	*Alangium salviifolium* (L.f.) Wangerin (sage-leaf alangium)	Cornaceae	S	229 ± 14	4.1 ± 0.1	4.0 ± 0.0	3.6 ± 0.1	1544 ± 31	308 ± 7
36	*Benincasa hispida* (Thunb.) Cogn.	Cucurbitaceae	V	63.80 ± 5.00	4.2 ± 0.1	47 ± 1	1.8 ± 0.4	448 ± 9	228 ± 9
37	*Citrullus lanatus* var. *lanatus* (Thunb.) Matsum. & Nakai	Cucurbitaceae	V	38.40 ± 2.00	4.2 ± 0.1	49 ± 1	2.9 ± 0.1	911 ± 19	728 ± 15
38	*Cucumis melo* var. *flexuosus* (L.) Naudin	Cucurbitaceae	V	19.9 ± 3.00	4.5 ± 0.1	33 ± 1	2.3 ± 0.1	361 ± 7	236 ± 5
39	*Cucumis melo* var. *Cantalupo* Ser.	Cucurbitaceae	V	25.30 ± 2.50	7.3 ± 0.2	28 ± 1	1.9 ± 0.4	437 ± 9	340 ± 8
40	*Cucumis sativus* L.	Cucurbitaceae	V	23.40 ± 1.90	4.7 ± 0.1	35 ± 1	1.7 ± 0.1	442 ± 8	356 ± 10
41	*Cucurbita maxima* Duchesne	Cucurbitaceae	V	132 ± 21	4.4 ± 0.1	18 ± 1	1.5 ± 0.0	542 ± 11	110 ± 3
42	*Diplocyclos palmatus* (L.) C. Reffrey	Cucurbitaceae	V	86.20 ± 4.70	4.9 ± 0.1	44 ± 1	1.6 ± 0.1	504 ± 11	304 ± 6
43	*Lagenaria siceraria* (Molina) Standl.	Cucurbitaceae	V	216 ± 5	4.9 ± 0.1	42 ± 1	1.7 ± 0.1	1316 ± 27	1140 ± 24
44	*Luffa acutangula* (L.) Roxb	Cucurbitaceae	V	122 ± 12	3.9 ± 0.1	47 ± 1	1.5 ± 0.0	235 ± 5	138 ± 3
45	*Luffa aegyptiaca* Mill.	Cucurbitaceae	V	105 ± 3	3.2 ± 0.1	43 ± 1	1.4 ± 0.0	306 ± 6	252 ± 5
46	*Momordica charantia* L. (big karela)	Cucurbitaceae	V	189 ± 13	4.7 ± 0.1	35 ± 1	1.8 ± 0.1	195 ± 4	118 ± 3
47	*Momordica charantia* L. (small karela)	Cucurbitaceae	V	102 ± 3	5.8 ± 0.1	30 ± 1	1.6 ± 0.1	3394 ± 67	170 ± 4
48	*Praecitrullus fistulosus* (Stocks) Pangalo	Cucurbitaceae	V	115 ± 3	5.0 ± 0.1	45 ± 1	1.8 ± 0.0	1250 ± 23	466 ± 14
49	*Solena amplexicaulis* (Lam.) Gandhi	Cucurbitaceae	V	178 ± 6	4.8 ± 0.1	32 ± 1	1.9 ± 0.1	4213 ± 81	390 ± 8
50	*Shorea robusta* C.F.Gaertn.	Dipterocarpaceae	T	995 ± 15	9.5 ± 0.1	20 ± 1	4.5 ± 0.1	3157 ± 62	2794 ± 55
51	*Diospyros melanoxylon* Roxb.	Ebenaceae	T	1310 ± 22	3.6 ± 0.1	-	3.5 ± 0.1	3546 ± 70	810 ± 9
52	*Jatropha curcas* L.	Euphorbiaceae	S	758 ± 15	7.5 ± 0.1	47 ± 1	5.5 ± 0.1	495 ± 6	426 ± 9
53	*Ricinus communis* L.	Euphorbiaceae	S	621 ± 13	3.5 ± 0.2	21 ± 1	3.5 ± 0.1	722 ± 15	646 ± 13
54	*Acacia auriculiformis* Benth.	Fabaceae	T	33.00 ± 2.00	5.6 ± 0.4	35 ± 1	3.6 ± 0.1	431 ± 9	380 ± 8
55	*Acacia catechu* (L.f.) Willd.	Fabaceae	T	57.00 ± 3.00	6.2 ± 0.5	45 ± 1	3.4 ± 0.1	954 ± 19	365 ± 7
56	*Acacia concinna* (Willd.) DC.	Fabaceae	T	224 ± 5	9.1 ± 0.6	39 ± 1	3.5 ± 0.1	715 ± 15	300 ± 6
57	*Acacia nilotica* Schumach. & Thonn.	Fabaceae	T	198 ± 4	7.8 ± 0.5	59 ± 1	3.8 ± 0.1	257 ± 5	255 ± 5
58	*Albizia saman* (Jacq.) Merr.	Fabaceae	T	219 ± 5	2.5 ± 0.1	42 ± 1	2.5 ± 0.1	518 ± 11	460 ± 13
59	*Albizia lebbek* (L.) Benth	Fabaceae	T	74.30 ± 2.00	7.1 ± 0.2	43 ± 1	3.1 ± 0.1	693 ± 14	405 ± 9
60	*Albizia odoratissima* (L.f.) Benth	Fabaceae	T	159 ± 3	2.5 ± 0.1	42 ± 1	2.5 ± 0.1	474 ± 15	430 ± 10
61	*Bauhinia purpurea* L.	Fabaceae	T	360 ± 11	6.2 ± 0.1	30 ± 1	3.1 ± 0.1	1214 ± 22	455 ± 9
62	*Bauhinia racemosa* Lam.	Fabaceae	T	173 ± 4	5.3 ± 0.1	72 ± 2	3.5 ± 0.1	2353 ± 47	415 ± 8
63	*Bauhinia vahlii* Wight & Arn.	Fabaceae	T	1855 ± 39	7.5 ± 0.2	44 ± 1	4.1 ± 0.1	293 ± 6	270 ± 6
64	*Butea frondosa* Willd.	Fabaceae	T	1003 ± 21	5.3 ± 0.1	-	4.5 ± 0.1	1816 ± 19	410 ± 9
65	*Caesalpinia decapetala* (Roth) Alston	Fabaceae	T	320 ± 10	6.5 ± 0.2	37 ± 1	3.1 ± 0.1	386 ± 8	350 ± 7
66	*Caesalpinia pulcherrima* (L.) Sw.	Fabaceae	S	38.00 ± 1.00	2.5 ± 0.1	63 ± 2	2.5 ± 0.1	2813 ± 56	2100 ± 41
67	*Cassia fistula* L. (golden shower)	Fabaceae	T	273 ± 5	4.1 ± 0.1	77 ± 2	2.1 ± 0.1	823 ± 17	460 ± 9
68	*Pithecellobium dulce* (Roxb.) Benth.	Fabaceae	T	164 ± 4	4.9 ± 0.1	25 ± 1	3.5 ± 0.1	386 ± 8	340 ± 7
69	*Pongamia pinnata* (L.) Pierre	Fabaceae	T	1431 ± 30	4.5 ± 0.1	5.0 ± 0.1	2.5 ± 0.1	477 ± 10	410 ± 9
70	*Saraca asoca* (Roxb.) Willd.	Fabaceae	T	800 ± 17	7.1 ± 0.2	15 ± 0	5.5 ± 0.1	1610 ± 31	1390 ± 28
71	*Sesbania grandiflora* (L.) Pers.	Fabaceae	T	85.30 ± 1.50	3.1 ± 0.1	35 ± 1	4.2 ± 0.1	1774 ± 34	550 ± 11
72	*Hardwickia binata* Roxb.	Fabaceae	T	294 ± 5	3.1 ± 0.1	-	2.2 ± 0.1	3494 ± 70	580 ± 12
73	*Pterocarpus marsupium* Roxb.	Fabaceae	T	880 ± 17	4.4 ± 0.1	56 ± 1	2.1 ± 0.1	2895 ± 57	340 ± 7
74	*Tamarindus indica* L.	Fabaceae	T	977 ± 20	6.5 ± 0.2	15 ± 0.	3.5 ± 0.1	299 ± 3	265 ± 6
75	*Sesbania sesban* (L.) Merr.	Fabaceae	S	13.80 ± 0.20	1.0 ± 0.0	-	-	2234 ± 45	500 ± 10
76	*Enterolobium cyclocarpum* (Jacq.) Griseb.	Fabaceae	T	875 ± 4	6.5 ± 0.2	45 ± 1	4.2 ± 0.1	418 ± 9	325 ± 7
77	*Gliricidia maculata* (Kunth) Steud.	Fabaceae	T	130 ± 4	4.5 ± 0.1	18 ± 0	2.5 ± 0.1	1021 ± 21	360 ± 7
78	*Delonix regia* (Bojer) Raf.	Fabaceae	T	510 ± 5	7.5 ± 0.2	69 ± 2	2.2 ± 0.1	382 ± 8	285 ± 5
79	*Entada gigas* (L.) Fawc. & Rendle	Fabaceae	V	23623 ± 2	8.3 ± 0.2	40 ± 1	1.5 ± 0.0	1884 ± 17	265 ± 5
80	*Leucaena leucocephala* (Lam.) de Wit	Fabaceae	S	61.30 ± 0.50	6.5 ± 0.2	47 ± 1	1.6 ± 0.0	1243 ± 25	320 ± 7
81	*Mimosa pudica* L.	Fabaceae	V	21.30 ± 0.50	3.2 ± 0.1	-	1.0 ± 0.0	1846 ± 17	625 ± 13
82	*Parkia javanica* Merr.	Fabaceae	T	305 ± 5	6.5 ± 0.2	37 ± 1	1.5 ± 0.0	988 ± 10	910 ± 18
83	*Senna siamea* (Lam.) H.S.Irwin & Barneby	Fabaceae	T	22.00 ± 1.00	3.6 ± 0.1	-	1.2 ± 0.0	418 ± 9	305 ± 6
84	*Juglans regia* L.	Juglandaceae	T	12200 ± 238	2.9 ± 0.1	32 ± 1	3.3 ± 0.1	182 ± 4	152 ± 3
85	*Litsea glutinosa* (Lour.) C.B.Rob.	Lauraceae	T	248 ± 6	3.5 ± 0.1	43 ± 1	3.8 ± 0.1	1836 ± 17	1588 ± 29
86	*Linum usitatissimum* L.	Linaceae	H	7.20 ± 0.10	4.9 ± 0.1	-	2.8 ± 0.1	332 ± 6	218 ± 5
87	*Strychnos potatorum* L.f.	Loganiaceae	T	283 ± 5	4.8 ± 0.1	24 ± 1	4.1 ± 0.1	1068 ± 21	964 ± 20
88	*Lagerstroemia parviflora* Roxb.	Lythraceae	T	2669 ± 50	5.2 ± 0.1	53 ± 1	6.5 ± 0.2	2350 ± 48	1060 ± 21
89	*Lawsonia inermis* L.	Lythraceae	S	22.00 ± 0.50	1.5 ± 0.0	-	2.1 ± 0.1	1990 ± 38	1088 ± 21
90	*Trapa natans* L.	Lythraceae	H	4923 ± 100	8.8 ± 0.2	15 ± 0	4.3 ± 0.1	1595 ± 31	1360 ± 26
91	*Careya arborea* Roxb.	Lecythidaceae	T	423 ± 9	6.5 ± 0.2	35 ± 1	2.5 ± 0.1	2607 ± 53	2280 ± 45
92	*Azadirachta indica* A.Juss.	Meliaceae	T	345 ± 7	6.1 ± 0.2	25 ± 1	3.5 ± 0.1	2438 ± 49	2162 ± 44
93	*Melia azedarach* L.	Meliaceae	T	972 ± 20	4.5 ± 0.1	40 ± 1	3.5 ± 0.1	3500 ± 71	3130 ± 63
94	*Abelmoschus esculentus* (L.) Moench	Malvaceae	H	59.80 ± 1.00	4.2 ± 0.1	-	4.8 ± 0.1	496 ± 9	444 ± 9
95	*Abelmoschus moschatus* (L.) Medik	Malvaceae	H	17.40 ± 0.30	4.1 ± 0.1	-	4.5 ± 0.1	2250 ± 41	1960 ± 19
96	*Abutilon indicum* (Link) Sweet	Malvaceae	H	5.43 ± 0.11	3.5 ± 0.1	-	4.3 ± 0.1	458 ± 9	436 ± 9
97	*Corchorus olitorius* L.	Malvaceae	H	2.08 ± 0.12	3.8 ± 0.1	-	2.7 ± 0.1	424 ± 9	312 ± 7
98	*Corchorus olitorius* L.	Malvaceae	H	1.05 ± 0.11	2.3 ± 0.1	-	2.6 ± 0.1	770 ± 15	668 ± 13
99	*Gossypium arboreum* L.	Malvaceae	H	74.40 ± 3.00	3.9 ± 0.1	-	3.8 ± 0.1	2009 ± 40	1786 ± 37
100	*Hibiscus cannabinus* L.	Malvaceae	H	22.90 ± 0.50	3.8 ± 0.1	-	3.3 ± 0.1	840 ± 9	730 ± 15
101	*Hibiscus sabdariffa* L.	Malvaceae	H	24.60 ± 0.50	2.6 ± 0.1	-	3.6 ± 0.1	508 ± 11	394 ± 8
102	*Malachra capitata* L.	Malvaceae	H	5.15 ± 0.14	2.9 ± 0.1	-	3.1 ± 0.1	271 ± 6	232 ± 7
103	*Sida acuta* Burm.f.	Malvaceae	H	2.38 ± 0.10	4.4 ± 0.1	-	3.5 ± 0.1	901 ± 18	734 ± 14
104	*Sida cordifolia* L.	Malvaceae	H	1.26 ± 0.11	7.6 ± 0.2	-	2.9 ± 0.1	2058 ± 41	854 ± 17
105	*Sterculia foetida* L.	Malvaceae	T	1244 ± 23	3.1 ± 0.1	-	1.5 ± 0.0	256 ± 5	152 ± 3
106	*Sterculia urens* Roxb.	Malvaceae	T	193 ± 4	2.5 ± 0.1	29 ± 1	1.5 ± 0.0	479 ± 8	422 ± 8
107	*Thespesia populnea* Sol. ex Corrêa	Malvaceae	T	162 ± 3	3.2 ± 0.1	47 ± 1	2.2 ± 0.0	2512 ± 52	2202 ± 43
108	*Urena lobata* L.	Malvaceae	V	15.27 ± 2.56	3.6 ± 0.1	-	4.2 ± 0.1	376 ± 8	322 ± 6
109	*Artocarpus heterophyllus* Lam.	Moraceae	T	10640 ± 34	30 ± 1.0	8.0 ± 0.2	6.1 ± 0.2	888 ± 18	316 ± 7
110	*Ficus racemosa* L.	Moraceae	T	2626 ± 51	8.5 ± 0.2	27 ± 1	2.5 ± 0.1	475 ± 10	414 ± 8
111	*Moringa oleifera* Lam.	Moringaceae	T	381 ± 8	6.5 ± 0.1	38 ± 1	3.5 ± 0.1	316 ± 7	264 ± 5
112	*Psidium guajava* L.	Myrtaceae	T	27.70 ± 0.50	2.5 ± 0.1	-	1.2 ± 0.0	296 ± 6	210 ± 4
113	*Syzygium cumini* (L.) Skeels	Myrtaceae	T	1280 ± 24	4.5 ± 0.1	25 ± 1	3.2 ± 0.1	2845 ± 58	2298 ± 45
114	*Nelumbo nucifera* Gaertn.	Nelumbonaceae	H	1213 ± 23	2.2 ± 0.1	12 ± 0	3.6 ± 0.1	2603 ± 54	428 ± 5
115	*Argemone mexicana* L.	Papeveraceae	H	2.03 ± 0.09	5.2 ± 0.1	-	1.7 ± 0.0	5000 ± 99	4465 ± 90
116	*Papaver somniferum* L.	Papeveraceae	H	0.21 ± 0.00	4.4 ± 0.1	-	1.3 ± 0.0	4307 ± 86	3890 ± 75
117	*Passiflora foetida* L.	Passifloraceae	H	7.13 ± 0.12	6.2 ± 0.1	-	2.1 ± 0.1	3622 ± 72	3170 ± 63
118	*Sesamum indicum* L.	Pedaliaceae	H	2.01 ± 0.03	3.5 ± 0.1	-	1.6 ± 0.0	2504 ± 49	2120 ± 40
119	*Sesamum radiatum* Schumach. & Thonn.	Pedaliaceae	H	1.07 ± 0.01	3.7 ± 0.1	-	1.5 ± 0.0	3398 ± 68	2975 ± 61
120	*Sesamum radiatum* Schumach. & Thonn.	Pedaliaceae	H	1.03 ± 0.00	3.8 ± 0.1	-	1.7 ± 0.0	4750 ± 97	4290 ± 85
121	*Piper nigrum* L.	Piperacee	H	37.00 ± 0.60	8.7 ± 0.2	-	7.8 ± 0.2	1949 ± 20	1660 ± 32
122	*Cleistanthus collinus* (Roxb.) Benth.	Phyllanthaceae	T	155 ± 3	4.5 ± 0.1	-	2.5 ± 0.1	2208 ± 44	675 ± 14
123	*Phyllanthus emblica* L.	Phyllanthaceae	T	921 ± 21	8.1 ± 0.2	95 ± 2	1.5 ± 0.0	493 ± 8	375 ± 8
124	*Bridelia retusa* A.Juss. (spinous kino tree)	Phyllanthaceae	T	154 ± 9	4.5 ± 0.1	19 ± 1	2.2 ± 0.1	3999 ± 82	3130 ± 63
125	*Persicaria punctate* Small	Polygonaceae	H	0.79 ± 0.10	2.1 ± 0.1	-	2.7 ± 0.1	1908 ± 39	770 ± 15
126	*Putranjiva roxburghii* Wall.	Putranjivaceae	T	478 ± 10	5.5 ± 0.1	66 ± 2	4.2 ± 0.1	493 ± 10	255 ± 5
127	*Nigella sativa* L.	Ranunculaceae	H	20.30 ± 2.15	5.5 ± 0.1	-	4.3 ± 0.1	4247 ± 86	3750 ± 39
128	*Ziziphus mauritiana* Lam.	Rhamnaceae	T	983 ± 22	11 ± 0.3	90 ± 2	4.5 ± 0.1	1279 ± 25	1065 ± 11
129	*Anthocephalus indicus* A.Rich.	Rubiaceae	T	20253 ± 43	36 ± 1.0	-	5.5 ± 0.1	1415 ± 27	570 ± 12
130	*Gardenia thunbergia* Thunb.	Rubiaceae	S	5480 ± 121	8.5 ± 0.2	37 ± 1	3.5 ± 0.1	607 ± 11	350 ± 7
131	*Prunus dulcis* (Mill.) D.A. Webb	Rosaceae	T	2623 ± 41	4.5 ± 0.1	29 ± 1	2.5 ± 0.1	598 ± 12	265 ± 7
132	*Aegle marmelos* L.	Rutaceae	T	148 ± 3	3.5 ± 0.1	22 ± 1	1.5 ± 0.0	1899 ± 17	1530 ± 31
133	*Citrus limon* (L.) Burm.f.	Rutaceae	S	68.30 ± 1.30	4.1 ± 0.1	17 ± 0	4.8 ± 0.2	567 ± 12	510 ± 11
134	*Citrus* × *sinensis* (L.) Osbeck	Rutaceae	S	120 ± 3	5.4 ± 0.1	22 ± 1	5.1 ± 0.2	663 ± 14	375 ± 8
135	*Murraya koenigii* Spreng.	Rutaceae	S	155 ± 3	2.9 ± 0.1	12 ± 0	1.5 ± 0.0	2927 ± 39	2520 ± 52
136	*Santalum album* L.	Santalaceae	T	183 ± 4	2.8 ± 0.1	40 ± 1	1.8 ± 0.1	1480 ± 31	1065 ± 22
137	*Cardiospermum halicacabum* L.	Sapindaceae	V	2.50 ± 0.20	3.1 ± 0.1	-	2.1 ± 0.1	779 ± 15	275 ± 6
138	*Litchi chinensis* Sonn.	Sapindaceae	T	2168 ± 41	11 ± 0.2	-	5.5 ± 0.0	2043 ± 42	410 ± 8
139	*Sapindus emarginatus* Vahl.	Sapindaceae	T	2148 ± 42	6.5 ± 0.1	61 ± 2	3.5 ± 0.1	342 ± 7	290 ± 6
140	*Schleichera oleosa* (Lour.) Oken	Sapindaceae	T	352 ± 7	4.1 ± 0.1	49 ± 1	3.6 ± 0.1	2442 ± 49	2195 ± 44
141	*Illicium verum* Hook.f.	Schisandraceae	H	26.70 ± 0.50	5.5 ± 0.1	58 ± 2	3.7 ± 0.0	1323 ± 26	565 ± 12
142	*Capsicum annuum* L. (small mirch)	Solanaceae	H	6.30 ± 0.15	4.7 ± 0.1	-	4.3 ± 0.0	460 ± 10	320 ± 6
143	*Capsicum annuum* L. (medium mirch)	Solanaceae	H	4.22 ± 0.10	4.3 ± 0.1	-	4.6 ± 0.1	419 ± 9	315 ± 7
144	*Datura stramonium* L.	Solanaceae	H	18.20 ± 0.40	6.5 ± 0.1	-	3.5 ± 0.1	935 ± 19	510 ± 11
145	*Solanum lycopersicum* L.	Solanaceae	H	2.01 ± 0.23	5.2 ± 0.1	-	5.3 ± 0.1	1298 ± 27	1080 ± 21
146	*Solanum melongena* L. (white)	Solanaceae	H	3.02 ± 0.05	4.5 ± 0.1	-	5.1 ± 0.1	728 ± 16	445 ± 9
147	*Solanum melongena* L. (purple)	Solanaceae	H	2.00 ± 0.04	6.4 ± 0.1	-	4.8 ± 0.1	865 ± 17	770 ± 15
148	*Solanum melongena* L. (green)	Solanaceae	H	3.01 ± 0.06	6.5 ± 0.1	-	5.2 ± 0.0	520 ± 11	455 ± 9
149	*Solanum melongena* L. (Singhi)	Solanaceae	H	2.01 ± 0.08	6.6 ± 0.1	-	4.9 ± 0.0	681 ± 13	470 ± 10
150	*Solanum virginianum* L.	Solenaceae	H	2.02 ± 0.05	6.3 ± 0.1	-	6.5 ± 0.0	2123 ± 43	680 ± 14
151	*Withania coagulans* Dunal	Solanaceae	H	399 ± 8	6.1 ± 0.1	15 ± 0	3.1 ± 0.0	944 ± 20	635 ± 13
152	*Withania somnifera* (L.) Dunal	Solanaceae	H	2.02 ± 0.06	1.4 ± 0.1	-	1.2 ± 0.0	837 ± 17	505 ± 10
153	*Lantana camara* L.	Verbenaceae	H	20.40 ± 0.05	8.3 ± 0.1	-	2.1 ± 0.1	614 ± 12	265 ± 6
154	*Amomum subulatum* Roxb.	Zingiberaceae	H	17.40 ± 0.06	4.3 ± 0.1	-	4.3 ± 0.1	427 ± 9	340 ± 7
155	*Elettaria cardamomum* Maton	Zingiberaceae	H	10.30 ± 0.20	5.1 ± 0.1	-	4.6 ± 0.0	298 ± 6	265 ± 5

H = Herb, S = Shrub, V = Vine, T = Tree.

**Table 2:** Mineral elements in cultivar with respect to plant type (mg/100 g).

Type	Na	Mg	Al	P	S	Cl	K	Ca	Ti	Cr	Mn	Fe	Co	Cu	Zn	Rb	Sr	Mo	Ba	Pb
T	286	169	0.9	349	435	21	1034	334	0.3	0.2	3.2	42	0.06	1.3	3.5	1.5	0.9	0.07	0.1	0.07
S	207	218	6.4	380	281	34	1239	368	1.3	0.2	3.9	107	0.02	1.1	5.2	1.4	1.4	0.12	0.0	0.07
H	137	206	10.3	449	327	80	974	499	1.0	0.1	6.9	35	0.07	1.4	4.3	1.6	2.5	0.01	0.5	0.05
V	0.0	156	0.0	496	226	9	581	156	0.2	0.0	5.5	20	0.02	1.5	5.5	1.3	0.6	0.01	0.1	0.03

H = Herb, T = Tree, V = Vine, S = Shrub.

## Data Availability

All data are included along the text in the corresponding tables.

## Supplementary Materials

The following supporting information can be downloaded at: https://www.mdpi.com/article/10.3390/molecules27103184/s1, Figure S1: Physical characteristics of seeds; Table S1: Mineral element concentration of seeds (mg/100 g); Table S2: Mineral element concentration in cultivars familywise (mg/100 g).

## Sample Availability

Original seeds are available upon request to Patel.
