# Supplementary material for: Seeds as Potential Sources of Phenolic Compounds and Minerals for the Indian Population
Source: Molecules. 2022 May 17;27(10):3184. doi: 10.3390/molecules27103184 (PMC9144825; doi:10.3390/molecules27103184)
Supplement: Supplementary file 1 [file molecules-27-03184-s001.zip › molecules-1709122-supplementary.pdf]

Seeds as Potential Sources of Phenolic Compounds and Minerals for Indian population

Supplemental information

Figure S1. Physical characteristics of seeds: (1) *Coriandrum sativum* L. (2) *Cuminum cyminum* L. (3) *Daucus carota* spp. *Sativus* (4) *Foeniculum vulgare* (5) *Trachyspermum ammi* (6) *Allium cepa* (7) *Asparagus racemosus* (8) *Helianthus annuus* L. (9) *Steria rebaudiana* (10) *Carthemnus oxycantha* L. (11) *Anacardium occidentale* (12) *Buchanania lanzan* (13) *Magnifera indica* (14) *Pistacia vera* L. (16) *Semcarpus anacardium* (17) *Annona squamosa* (18) *Areca catechu* (19) *Areca catechu* (20) *Phoenix dactylifera* (21) *Phoenix sylvestris* (22) *Basella rubra* L. (23) *Bixa orellana* (24) *Brassica campestris* (25) *Brassica hirta* (26) *Brassica nigra* (27) *Brassica oleracea* var. *Capitata F. Alba* (28) *Brassica oleracea* var. *Botrytis* (29) *Brassica rapa* (30) *Lepidium sativum* L. (31) *Raphanus sativus* (32) *Sisymbrium irio* L. (33) *Commiphora wightii* (34) *Carica papaya* (35) *Celastrus paniculatus* (36) *Allangium salviifolium* (37) *Benincasa hispida* (38) *Citrullus lanatus* Var. *Lanatus* (39) *Cucumis melo* Var *Flexuosus* (40) *Cucumis melo* var. *Cantalupo* (41) *Cucumis sativus* (42) *Cucurbita maxima* (43) *Diplocyclos plamatus* (44) *Lagenaria siceraria* (45) *Luffa acutangula* (46) *Luffa aegyptiaca* (47) *Momordica charantia* L.-I (48) *Momordica charantia* L.-II (49) *Praecitrullus fistulosus* (50) *Solena amplexicaulis* (51) *Shorea robusta* (52) *Diosporous melanaoxylen* (53) *Jatropha curcas* (54) *Ricius communis* (55) *Acacia auriculiformis* (56) *Acacia catechu* (57) *Acacia concinna* (58) *Acacia nilotica* (59) *Albizia saman* (60) *Albizzia lebbek* (61) *Albizzia odoratissima* (62) *Bauhinia pruperia* (63) *Bauhinia racemosa* (64) *Bauhinia vahlii* (65) *Butea frondosa* (66) *Caesalpinia decapetala* (67) *Caesalpinia pulcherrima* (68) *Cassia fistula* (69) *Pithecellobium dulce* (70) *Pongamia pinnata* (71) *Saraca asoca* (72) *Sesbenia grandiflora* (73) *Hardwickia binata* (74) *Pterocarpus marsupium* (75) *Tamarindus indica* (76) *Sesbenia sesben* (77) *Enterolobium cyclocarpum* (78) *Gliricidia maculata* (79) *Delonix regia* (80) *Entada gigas* (81) *Leucanea lecocephala* (82) *Mimosa pudica* (83) *Parkia javanica* (84) *Senna siamea* (85) *Juglans regia* L. (86) *Litsea glutinosa* (87) *Linum usitatissimum* L. (88) *Strychnos potatorum* (89) *Lagerstroemia parviflora* (90) *Lawsonia inermis* (91) *Trapa natans* (92) *Careya arborea* (93) *Azadirachta indica* (94) *Melia azedarach* (95) *Abelmoschus esculentus* (96) *Abelmoschus moschatus* (97) *Abutilon indicum* (98) *Corchorus olitorius* L.-I (99) *Corchorus olitorius* L.-II (100) *Gossypium arboreum* (101) *Hibiscus cannabinus* (102) *Hibiscus sabdariffa* (103) *Melachra capitata* (104) *Sida acuta* (105) *Sida cordifolia* (105) *Sterculia foetida* (106) *Sterculia urens* (107) *Thespesia populnea* (108) *Urena lobata* (109) *Artocarpus heterophyllus* (110) *Ficus racemosa* (111) *Moringa oleifera* (112) *Psidium guajava* (113) *Syzygium cumini* (114) *Nilumbo nucifera* (115) *Argemone mexicana* L. (116) *Papaver somniferum* (117) *Passiflora foetida* L. (118) *Sesamum indicum* (119) *Sesamum radiatum*-I (120) *Sesamum radiatum*-II (121) *Piper nigrum* (122) *Cleistanthus collinus* (123) *Phyllanthus emblica* L. (124) *Bridelia retusa* (125) *Persicaria punctata* (126) *Putranjiva roxburghii*, (127) *Nigella sativa*, (128) *Ziziphus auritiana*, (129) *Anthocephalus indicus* (130) *Gardenia thunbergia* (131) *Prunus dulcis* (132) *Aegle marmelon* (133) *Citrus limon* (134) *Citrus sinensis* (135) *Murraya koenigii* (136) *Santalum album* (137) *Cardiospermum halicacabum* L. (138) *Litchi chinesis* (139) *Sapindus emerguinatus* (140) *Schleichera oleosa* (141) *Illicum verum* (142) *Capsicum annuum* L.-I (143) *Capsicum annuum* L.-II (144) *Datura stramonium* (145) *Solanum lycopersicum* (146) *Solanum melongena*-I (147) *Solanum melongena*-II (148) *Solanum melongena*-III (149) *Solanum melongena*-IV (150) *Solanum virginianum* (151) *Withania coagulans* (152) *Withania somnifera* (153) *Lantana camara* (154) *Amomum subulatum* (155) *Elettaria cardamomum*.

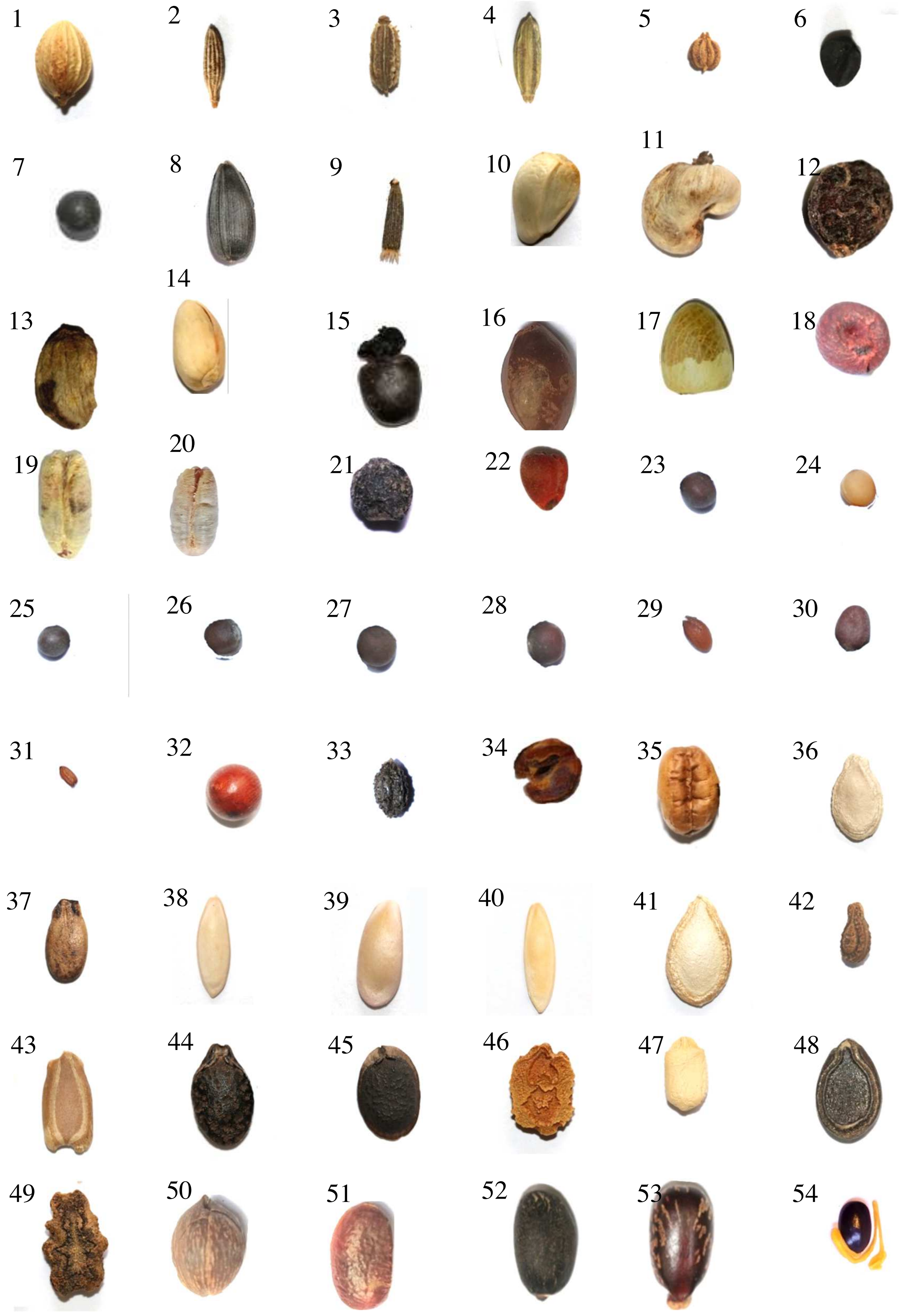
1
2
3
4
5
6
7
8
9
10
11
12
13
14
15
16
17
18
19
20
21
22
23
24
25
26
27
28
29
30
31
32
33
34
35
36
37
38
39
40
41
42
43
44
45
46
47
48
49
50
51
52
53
54

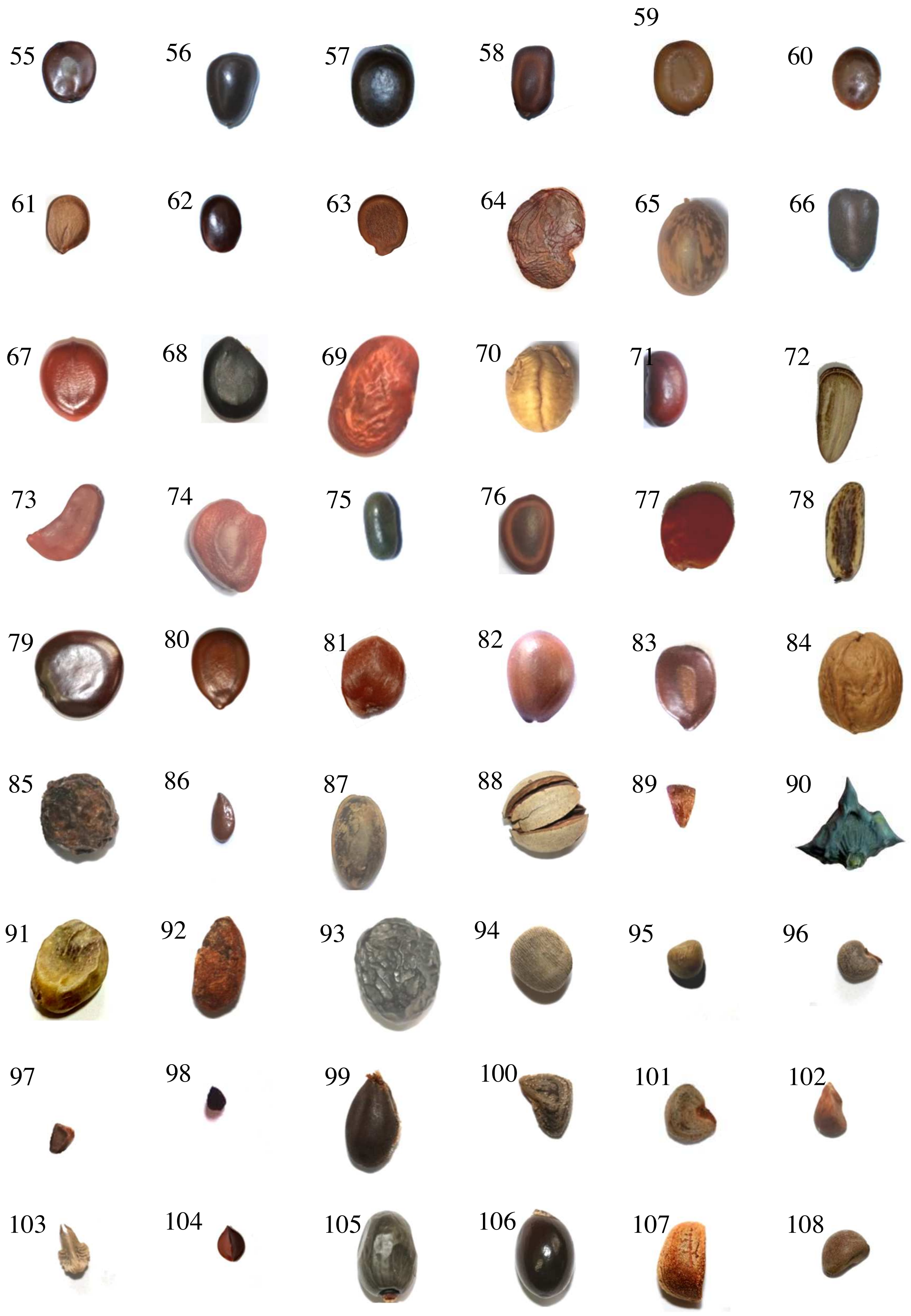
55
56
57
58
59
60
61
62
63
64
65
66
67
68
69
70
71
72
73
74
75
76
77
78
79
80
81
82
83
84
85
86
87
88
89
90
91
92
93
94
95
96
97
98
99
100
101
102
103
104
105
106
107
108

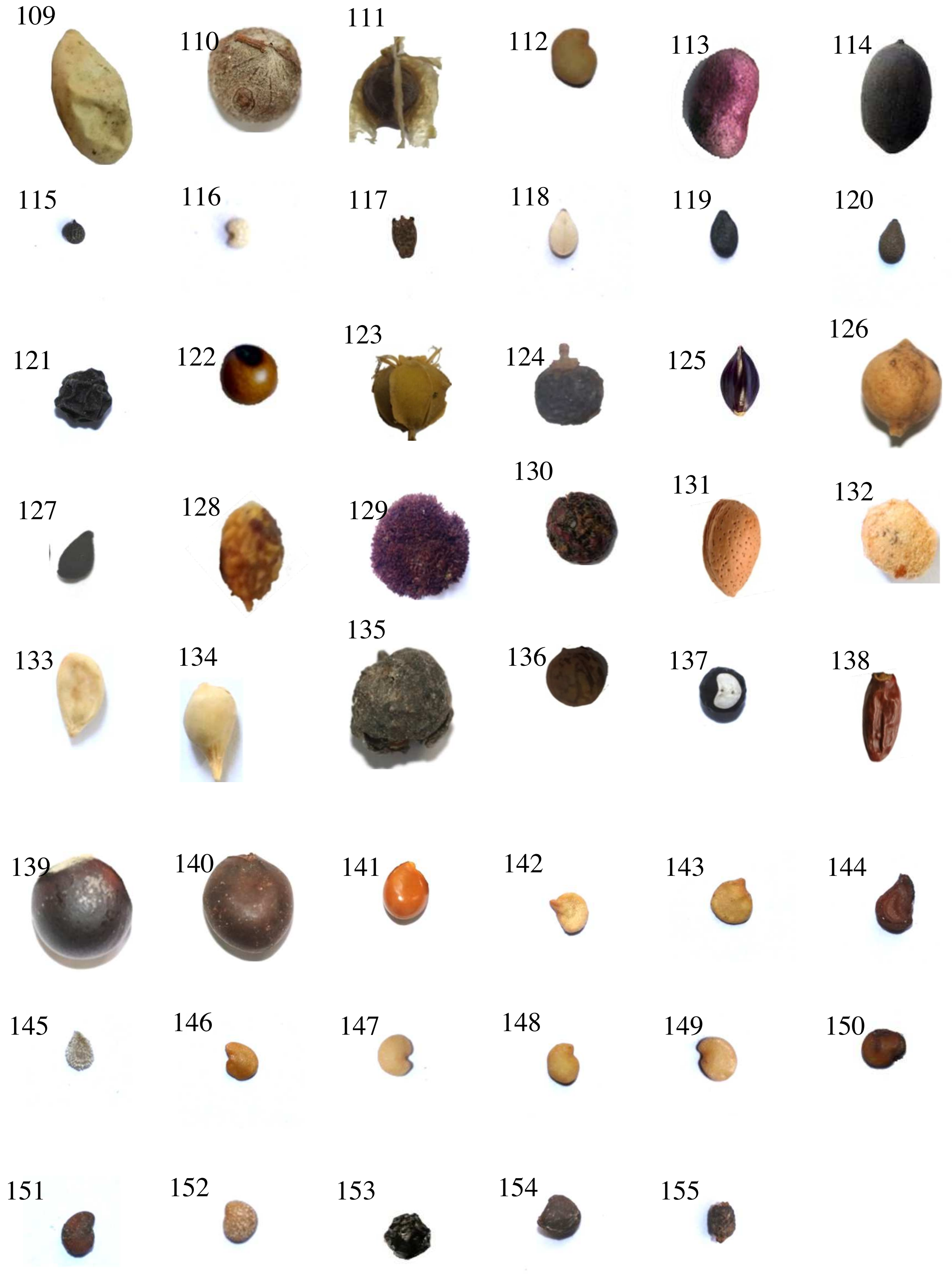
109
110
111
112
113
114
115
116
117
118
119
120
121
122
123
124
125
126
127
128
129
130
131
132
133
134
135
136
137
138
139
140
141
142
143
144
145
146
147
148
149
150
151
152
153
154
155

Table S1. Mineral elements concentration of seeds (mg/100g).

Nº	Na	Mg	Al	P	S	Cl	K	Ca	Ti	Cr	Mn	Fe	Co	Cu	Zn	Rb	Sr	Mo	Ba	Pb
1	BDL	219	BDL	244	178	520	1985	514	1.3	BDL	6.2	21.8	BDL	1.7	3.0	3.1	2.8	BDL	BDL	0.2
2	1403	385	BDL	174	330	764	2417	1200	2.1	BDL	7.0	29.9	0.1	0.9	4.3	1.6	7.8	BDL	BDL	BDL
3	BDL	272	21	392	361	463	1336	1621	13.2	BDL	8.8	36.5	0.2	1.5	6.0	1.2	27.4	BDL	7.9	0.2
4	BDL	262	BDL	224	315	675	2411	1786	1.2	BDL	10.9	13.3	0.1	1.3	4.0	2.9	20.5	BDL	1.6	0.1
5	BDL	137	240	209	210	139	1523	1143	13.9	0.4	10.3	303	BDL	1.0	6.6	4.3	8.6	BDL	2.3	0.1
6	BDL	328	BDL	532	579	BDL	890	520	1.7	BDL	5.7	31.5	BDL	1.0	5.2	0.7	0.9	BDL	BDL	0.1
7	BDL	117	BDL	215	216	BDL	388	142	BDL	0.5	1.5	15	BDL	1.0	1.7	0.6	BDL	BDL	BDL	BDL
8	BDL	154	BDL	498	207	BDL	786	279	BDL	BDL	3.5	15.7	BDL	1.9	6.9	1.3	0.4	BDL	BDL	BDL
9	BDL	238	BDL	606	211	BDL	780	962	1.8	BDL	4.5	47.6	BDL	0.8	2.9	0.9	2.6	BDL	1.4	BDL
10	BDL	177	BDL	537	161	13	894	240	BDL	BDL	3.3	110	BDL	1.8	5.3	1.1	0.8	BDL	BDL	BDL
11	BDL	107	BDL	425	197	BDL	980	120	BDL	BDL	2.0	11	BDL	1.5	8.7	2.3	BDL	BDL	BDL	BDL
12	BDL	70	BDL	321	162	BDL	352	49	BDL	BDL	2.8	18	BDL	0.7	4.7	2.8	0.5	BDL	BDL	BDL
13	BDL	BDL	BDL	139	61	BDL	1045	93	BDL	0.5	0.8	10.2	BDL	8.7	BDL	3.2	0.1	BDL	BDL	0.1
14	BDL	BDL	BDL	236	122	479	582	54	BDL	BDL	0.6	3.8	0.1	0.8	1.4	1.4	0.3	BDL	BDL	BDL
15	BDL	39	BDL	133	56	BDL	411	176	BDL	BDL	6.2	5.1	0.1	0.5	1.4	0.8	1.6	BDL	BDL	BDL
16	BDL	46	BDL	120	294	73	557	54	BDL	0.4	3.7	233	BDL	1.7	BDL	1.1	0.1	BDL	BDL	0.3
17	BDL	46	BDL	120	294	73	557	54	BDL	0.4	3.7	233	BDL	1.7	BDL	1.1	0.1	BDL	BDL	0.3
18	BDL	114	BDL	181	881	171	818	134	BDL	0.3	5.3	137	BDL	0.9	1.0	2.1	BDL	BDL	BDL	0.2
19	BDL	99	BDL	96	162	BDL	351	144	2.4	0.7	7.5	510	BDL	0.7	2.0	0.4	BDL	BDL	BDL	0.4
20	2319	43	BDL	101	197	BDL	315	39	1.2	0.9	5.9	499	0.1	0.6	2.5	0.2	BDL	BDL	BDL	0.5
21	BDL	262	BDL	539	269	176	1431	19	2.5	BDL	5.9	42.6	BDL	0.6	10.3	0.7	0.2	BDL	BDL	0.1
22	BDL	143	BDL	311	211	BDL	1371	264	BDL	BDL	5.2	68.5	BDL	0.6	2.1	2.8	0.9	BDL	BDL	0.1
23	BDL	190	BDL	539	930	BDL	754	584	BDL	BDL	5.3	43.2	BDL	0.7	5.3	0.9	2.2	BDL	BDL	BDL
24	BDL	124	BDL	415	710	BDL	550	418	BDL	BDL	4.3	10.3	0.1	0.3	3.9	1.2	3.2	BDL	BDL	BDL
25	BDL	161	BDL	423	733	BDL	567	411	1.8	BDL	5.9	52.3	BDL	0.5	3.9	1.8	1.5	BDL	BDL	BDL
26	BDL	212	BDL	609	793	BDL	603	846	BDL	BDL	4.9	13.8	0.1	0.3	4.9	0.3	1.3	BDL	1.0	BDL
27	BDL	288	BDL	590	1493	BDL	1029	434	BDL	BDL	4.4	48.8	BDL	0.5	10.7	1.2	2.5	BDL	BDL	BDL
28	BDL	191	BDL	536	819	BDL	962	444	BDL	BDL	5.0	30	BDL	0.6	4.0	1.4	2.6	BDL	BDL	BDL
29	BDL	204	BDL	433	875	BDL	1279	407	BDL	BDL	3.4	15.4	BDL	0.6	6.2	13.4	0.4	BDL	BDL	0.2
30	BDL	273	BDL	616	1478	BDL	963	356	BDL	BDL	4.9	19.3	0.1	0.5	6.1	1.0	1.4	BDL	BDL	BDL
31	BDL	169	14	492	505	BDL	775	571	2.9	BDL	8.7	72.1	BDL	0.8	5.8	1.3	1.2	BDL	BDL	0.1
32	2065	910	67	95	73	253	1022	584	8.7	1.6	8.4	1100	BDL	0.5	20.7	1.1	2.8	0.3	BDL	0.3
33	BDL	374	BDL	589	782	134	1927	929	2.7	BDL	9.3	37.9	BDL	1.0	7.5	3.5	2.9	0.2	BDL	0.2
34	BDL	38	BDL	149	82	BDL	530	372	1.6	BDL	3.7	26.8	BDL	0.7	1.1	1.5	3.2	BDL	1.2	0.1
35	BDL	95	BDL	303	172	BDL	1539	40	BDL	BDL	1.1	22	BDL	1.2	2.4	1.4	BDL	BDL	BDL	BDL
36	BDL	103	BDL	525	197	BDL	445	42	BDL	BDL	9.1	14.6	BDL	1.4	7.4	2.0	BDL	BDL	BDL	BDL
37	BDL	95	BDL	481	220	BDL	477	24	BDL	BDL	4.7	15.3	BDL	1.2	5.8	1.9	BDL	BDL	BDL	BDL
38	BDL	201	BDL	669	276	BDL	605	53	BDL	BDL	7.2	28.9	BDL	2.2	6.5	3.4	0.2	BDL	BDL	0.1
39	BDL	252	BDL	726	297	BDL	698	54	BDL	BDL	5.7	45.9	BDL	2.0	9.0	3.3	BDL	BDL	BDL	0.1
40	BDL	252	BDL	707	293	BDL	713	63	BDL	BDL	6.9	21.7	BDL	1.9	8.8	2.1	BDL	BDL	BDL	BDL
41	BDL	113	BDL	550	161	BDL	392	BDL	BDL	BDL	6.3	15.7	BDL	1.4	7.8	1.1	BDL	BDL	BDL	BDL
42	BDL	300	BDL	637	263	BDL	473	611	BDL	BDL	6.3	17.4	0.1	1.4	5.2	0.4	2.6	BDL	BDL	BDL
43	BDL	43	BDL	329	99	BDL	184	4.0	BDL	0.4	4.1	16.8	0.1	3.5	2.0	0.9	BDL	BDL	BDL	BDL
44	BDL	148	BDL	601	202	BDL	615	37	BDL	0.5	3.7	27.9	BDL	2.5	7.0	1.2	BDL	BDL	BDL	BDL
45	BDL	246	BDL	703	239	BDL	928	9.0	BDL	BDL	3.0	9.9	0.1	1.7	7.0	1.3	BDL	BDL	BAL	PBL
46	BDL	72	BDL	366	162	BDL	266	3.0	BDL	BDL	1.7	14	BDL	0.4	5.6	0.8	BDL	BDL	BDL	BDL
47	BDL	52	BDL	339	142	BDL	214	17	BDL	BDL	1.7	13.7	BDL	0.7	3.8	0.6	BDL	BDL	BDL	BDL
48	BDL	121	BDL	543	197	BDL	449	97	BDL	BDL	0.9	10.1	BDL	1	5.9	0.9	0.1	BDL	BDL	BDL
49	BDL	49	BDL	323	101	BDL	201	BDL	BDL	BDL	2.6	12.5	BDL	1.2	4.9	0.2	BDL	BDL	BDL	BDL
50	BDL	50	BDL	138	91	BDL	649	80	BDL	BDL	5.5	2.7	BDL	0.8	1	0.7	0.5	BDL	BDL	BDL
51	BDL	49	BDL	53	50	BDL	205	508	BDL	BDL	1.1	80	BDL	0.6	0.9	0.5	2.2	BDL	BDL	0.1
52	BDL	191	832	551	160	832	803	251	BDL	BDL	2.6	9.6	BDL	1.0	4.2	1.6	0.2	BDL	BDL	BDL
53	832	247	BDL	585	263	BDL	558	321	BDL	BDL	BDL	8.2	BDL	1.0	8	0.2	BDL	BDL	BDL	BDL
54	BDL	254	BDL	249	518	BDL	875	780	1.3	BDL	12.5	33.9	BDL	0.6	2.8	2.5	0.7	BDL	BDL	BDL
55	BDL	366	BDL	524	1432	68	1421	745	BDL	BDL	3.1	10.3	0.1	0.8	4.6	1.6	2.9	BDL	BDL	BDL

56	BDL	252	BDL	362	247	BDL	835	643	BDL	BDL	3.3	13.2	0.1	0.9	2.6	1.0	0.6	0.1	BDL	BDL
57	BDL	395	BDL	641	2171	BDL	1503	344	BDL	BDL	3.3	16.8	0.1	1.6	10.9	2.0	0.9	BDL	BDL	BDL
58	BDL	136	BDL	441	384	161	1003	133	BDL	BDL	5.3	45.8	BDL	1.0	2.9	1.0	0.2	0.3	BDL	0.2
59	BDL	283	BDL	422	693	79	1591	649	BDL	0.4	5.9	25	0.1	1.2	5.2	1.0	0.6	BDL	BDL	BDL
60	BDL	226	BDL	378	655	BDL	993	626	BDL	0.5	4.2	15.2	0.1	1.3	2.4	2.5	0.6	0.2	BDL	BDL
61	BDL	240	BDL	503	238	BDL	1047	460	BDL	BDL	2.7	19.3	0.1	2.6	3.9	1.5	1.8	BDL	BDL	BDL
62	BDL	330	BDL	573	219	BDL	964	284	BDL	0.5	3.8	19.3	0.1	1.8	3.7	1.3	0.6	BDL	BDL	0.2
63	BDL	229	BDL	431	155	BDL	896	256	BDL	3.3	3.1	32.2	BDL	1.3	2.2	1.0	0.9	0.2	BDL	BDL
64	BDL	273	BDL	347	288	BDL	2579	411	1.8	BDL	4.5	10.8	0.1	2.3	6.7	1.8	0.6	BDL	BDL	BDL
65	BDL	129	BDL	462	217	BDL	1142	485	BDL	0.5	1.8	14.2	0.1	0.7	4.2	1.2	1.2	BDL	BDL	0.1
66	BDL	286	BDL	599	363	BDL	1222	540	BDL	BDL	2.0	8.5	0.1	1.3	5.1	1.5	2.5	BDL	BDL	BDL
67	BDL	415	BDL	561	217	BDL	1296	853	BDL	BDL	5.7	22.3	BDL	0.9	6.5	0.8	0.8	BDL	BDL	0.4
68	BDL	130	BDL	334	1076	BDL	952	35	BDL	0.6	4.2	15.9	0.1	0.7	3.6	0.8	BDL	BDL	BDL	BDL
69	BDL	43	BDL	204	143	BDL	639	469	BDL	0.4	5.9	6.9	0.1	0.7	3.2	2.1	0.6	BDL	BDL	BDL
70	BDL	101	BDL	226	148	BDL	1834	434	BDL	BDL	3.3	12.9	0.1	2.3	0.6	2.6	0.7	BDL	BDL	0.1
71	BDL	239	BDL	912	213	BDL	1474	157	BDL	BDL	2.6	13.9	BDL	0.7	8.6	2.9	1.3	2.7	BDL	0.1
72	BDL	152	BDL	212	160	BDL	755	595	BDL	BDL	1.1	7.1	0.1	1.4	4.9	1.2	1.6	BDL	1.0	BDL
73	BDL	556	BDL	674	599	BDL	1712	509	BDL	BDL	4.1	14.5	BDL	3.9	5.0	2.0	1.1	BDL	BDL	BDL
74	2378	122	BDL	177	125	BDL	522	234	BDL	BDL	BDL	7.5	BDL	1.0	2.5	0.3	0.3	BDL	BDL	BDL
75	BDL	112	BDL	670	302	BDL	1224	196	BDL	BDL	5.6	24.9	BDL	3.3	9.6	1.1	BDL	1.2	BDL	0.2
76	BDL	295	BDL	516	210	BDL	975	23	BDL	BDL	1.5	12.4	BDL	0.8	3.0	1.6	BDL	0.4	BDL	0.1
77	BDL	240	BDL	694	435	BDL	1070	195	BDL	BDL	3.9	13.4	0.1	0.5	5.0	3.1	0.2	BDL	BDL	BDL
78	BDL	278	BDL	681	447	BDL	2020	552	BDL	BDL	13.9	26.2	0.1	2.1	7.5	2.2	0.3	0.3	BDL	0.2
79	BDL	141	BDL	253	527	BDL	990	102	BDL	BDL	0.9	5.4	BDL	1.3	2.5	2.4	BDL	0.1	BDL	BDL
80	BDL	343	BDL	556	1083	36	1954	379	BDL	BDL	1.5	15.6	0.1	1.8	4.6	0.8	0.8	BDL	BDL	BDL
81	BDL	236	BDL	568	331	BDL	533	712	BDL	BDL	23.3	50.7	BDL	2.9	1.0	0.4	4.7	BDL	BDL	0.2
82	BDL	592	56	300	5144	BDL	774	1016	BDL	BDL	3.0	9.9	0.1	1.3	3.9	1.0	3.2	BDL	BDL	BDL
83	BDL	346	BDL	730	394	BDL	1264	1524	BDL	BDL	2.2	13.3	0.1	1.5	6.7	2.4	13.2	0.2	2.2	BDL
84	BDL	BDL	BDL	233	71	BDL	349	64	0.9	BDL	1.0	4.5	BDL	1.0	2.1	0.4	0.3	BDL	BDL	BDL
85	BDL	121	BDL	373	327	BDL	890	198	BDL	BDL	4.6	6.5	0.1	1.3	4.2	1.0	0.5	BDL	BDL	BDL
86	BDL	151	BDL	273	154	BDL	622	361	BDL	BDL	3.4	8.6	0.1	0.9	5.5	0.6	0.3	BDL	BDL	BDL
87	4305	164	BDL	368	252	BDL	784	262	BDL	BDL	1.6	4.7	0.1	0.8	3.6	2.3	0.3	BDL	BDL	BDL
88	BDL	135	BDL	309	191	BDL	2527	187	1.8	0.5	3.0	162	BDL	2.3	2.8	4.8	0.3	BDL	BDL	0.2
89	BDL	230	BDL	358	165	59	1626	702	3.7	BDL	4.7	58.7	BDL	1.0	2.0	1.8	3.7	BDL	BDL	0.1
90	78	BDL	269	87	121	1079	21	BDL	BDL	0.8	4.8	0.1	0.3	2.8	BDL	BDL	BDL	BDL	BDL	0
91	BDL	210	BDL	437	536	BDL	703	137	BDL	0.5	1.7	13.5	0.1	4.2	2.7	1.3	0.3	BDL	BDL	0.2
92	BDL	119	BDL	305	665	BDL	1397	147	1.5	BDL	1.8	13.7	BDL	1.0	4.5	0.7	0.7	BDL	BDL	BDL
93	BDL	142	BDL	464	240	BDL	816	203	BDL	BDL	1.3	17.6	BDL	1.0	6.5	1.4	0.1	BDL	BDL	BDL
94	BDL	406	BDL	905	355	BDL	1710	186	BDL	BDL	3.7	12.6	0.1	2.2	11.6	1.2	0.5	BDL	BDL	BDL
95	BDL	363	BDL	715	283	BDL	942	752	BDL	BDL	5.0	6.3	0.1	0.9	4.2	0.6	0.7	BDL	BDL	BDL
96	1709	317	BDL	696	317	BDL	1324	351	0.9	BDL	3.3	14.7	0.1	1.0	6.8	0.8	0.5	BDL	BDL	BDL
97	BDL	350	BDL	774	193	BDL	1210	941	BDL	BDL	3.3	28.9	BDL	1.7	2.9	1.3	2.7	BDL	BDL	BDL
98	BDL	472	BDL	958	265	BDL	1146	1546	BDL	BDL	2.2	12.1	0.1	2.3	3.3	0.2	3.8	0.1	1.8	BDL
99	BDL	333	BDL	798	313	BDL	1339	183	BDL	BDL	2.6	15.8	0.1	1.4	5.4	0.2	0.7	BDL	BDL	BDL
100	BDL	337	BDL	752	292	BDL	1541	248	1.7	BDL	8.8	16.7	0.1	1.0	6.2	2.4	1.2	BDL	BDL	0.1
101	BDL	338	BDL	721	342	BDL	1577	287	1.0	BDL	5.8	9.4	0.1	1.2	7.0	2.0	0.4	BDL	BDL	BDL
102	902	314	BDL	594	336	BDL	895	1395	BDL	BDL	11	19.3	0.2	3.4	5.1	0.7	1.6	BDL	1.3	0.1
103	BDL	317	BDL	466	368	BDL	1457	929	BDL	BDL	12.3	34	0.1	12.6	0	2.7	2.0	BDL	BDL	0.2
104	2068	231	BDL	468	130	BDL	306	195	BDL	BDL	7.4	58.4	BDL	0.4	6.2	0.8	0.5	0.1	BDL	0.1
105	BDL	0	BDL	205	113	BDL	542	BDL	BDL	BDL	BDL	2.6	0.1	1.2	8.0	1.7	0.1	BDL	BDL	BDL
106	BDL	210	BDL	557	275	BDL	1020	25	BDL	BDL	1.9	8.8	0.1	1.9	7.0	1.7	0.2	BDL	BDL	BDL
107	BDL	219	BDL	648	236	BDL	1296	358	BDL	BDL	1.3	6.0	0.1	1.7	2.8	1.5	0.4	BDL	BDL	BDL
108	BDL	396	BDL	665	385	BDL	1030	643	BDL	BDL	11	8.4	0.1	1.9	7.1	1.1	1.0	BDL	BDL	BDL
109	BDL	262	BDL	356	235	108	2171	523	1.7	BDL	5.5	49	BDL	1.2	3.1	4.0	1.8	0.2	BDL	0.2
110	BDL	204	BDL	230	83	58	2625	683	3.4	BDL	2.1	21.3	0.1	0.6	0.9	2.0	1.7	BDL	BDL	BDL
111	BDL	317	BDL	602	2067	BDL	1006	118	BDL	BDL	2.3	8.4	0.1	0.6	4.3	0.5	0.1	BDL	BDL	BDL
112	BDL	102	BDL	312	199	BDL	316	60	BDL	BDL	1.3	38.2	BDL	1.2	1.4	0.5	BDL	BDL	BDL	BDL
113	1018	42	BDL	66	61	BDL	789	14	BDL	BDL	BDL	3.0	0.1	0.5	0.4	1.6	BDL	BDL	BDL	BDL

114	BDL	176	BDL	571	284	BDL	1310	201	BDL	0.3	7.9	19.2	BDL	2.0	3.5	3.6	1.7	BDL	BDL	0.1
115	BDL	57	BDL	263	106	BDL	625	577	BDL	BDL	2.3	14.5	0.1	1.6	3.1	0.8	2.7	BDL	BDL	BDL
116	BDL	85	BDL	419	175	BDL	338	1072	BDL	BDL	8.5	15.8	0.2	2.2	5.8	0.9	6.8	BDL	1.3	BDL
117	BDL	45	BDL	321	145	BDL	565	39	BDL	BDL	1.1	10.7	BDL	1.0	3.4	0.5	BDL	BDL	BDL	BDL
118	BDL	135	BDL	355	175	BDL	284	830	BDL	BDL	1.1	10.8	0.1	0.6	3.6	0.7	2.3	BDL	1.2	BDL
119	BDL	125	BDL	318	189	BDL	399	994	2.1	BDL	2.9	36.3	BDL	0.9	3.8	0.6	3.8	BDL	2.6	BDL
120	BDL	97	BDL	297	169	BDL	302	934	1.8	BDL	2.5	27.3	0.1	1.0	3.2	0.5	4.6	BDL	3.1	BDL
121	476	143	BDL	176	121	373	1924	467	BDL	BDL	6.1	10.1	0.1	1.8	BDL	4.6	2.3	BDL	BDL	0.2
122	BDL	53	BDL	147	64	BDL	1701	436	BDL	BDL	1.5	19.6	BDL	0.8	1.5	0.6	0.9	BDL	BDL	BDL
123	BDL	82	BDL	318	167	BDL	1093	157	BDL	BDL	1.0	6.2	0.1	1.3	1.1	2.1	0.4	BDL	BDL	0.1
124	BDL	53	BDL	147	64	BDL	1701	436	BDL	BDL	1.5	19.6	BDL	0.8	1.5	0.6	0.9	BDL	BDL	BDL
125	BDL	204	BDL	422	85	BDL	809	80	BDL	BDL	7.8	80.8	BDL	0.5	2.8	1.2	0.4	0.1	BDL	BDL
126	1290	242	BDL	259	1587	BDL	1324	1059	BDL	BDL	0.9	5.3	0.1	1.1	1.0	1.5	3.0	BDL	BDL	BDL
127	BDL	115	BDL	404	227	BDL	653	745	BDL	BDL	3.4	9	0.1	1.0	5.7	1.2	3.6	BDL	1.2	BDL
128	BDL	149	BDL	461	337	BDL	707	146	BDL	BDL	4.1	14.9	0.1	1.0	11.7	1.6	0.3	BDL	BDL	BDL
129	565	141	BDL	92	82	BDL	1717	607	1.4	BDL	3.5	38.9	BDL	0.8	1.3	0.2	2.0	0.1	BDL	0.2
130	BDL	0	BDL	59	53	BDL	1243	104	BDL	BDL	BDL	4.5	BDL	1.0	BDL	1.9	BDL	BDL	BDL	BDL
131	BDL	94	BDL	306	107	BDL	596	199	BDL	BDL	1.8	4.9	0.1	0.9	3.2	0.6	1.0	BDL	BDL	BDL
132	BDL	159	BDL	370	199	BDL	621	1105	BDL	BDL	1.5	12.3	0.2	0.7	2.2	0.6	4.1	BDL	1.3	BDL
133	BDL	67	BDL	264	162	BDL	485	399	1.6	0.7	10.1	120	BDL	0.5	1.8	1.1	1.4	BDL	BDL	0.1
134	BDL	0	BDL	241	92	BDL	1056	160	1.4	BDL	1.3	12.3	BDL	0.9	1.9	0.7	3.9	BDL	BDL	BDL
135	BDL	55	BDL	144	53	BDL	1313	289	BDL	BDL	1.0	11	0.1	0.8	1.5	0.4	0.5	BDL	BDL	BDL
136	6419	122	BDL	319	218	55	589	164	BDL	BDL	0.5	5.0	BDL	0.7	2.6	0.9	0.3	BDL	BDL	BDL
137	BDL	0	BDL	241	85	BDL	445	260	BDL	BDL	1.8	6.7	BDL	0.4	1.6	0.3	0.4	BDL	BDL	BDL
138	BDL	34	BDL	148	66	BDL	951	0	BDL	BDL	0	5.2	0.1	1.2	0.9	0.8	BDL	BDL	BDL	BDL
139	BDL	44	BDL	406	163	BDL	755	85	BDL	BDL	0.9	7.9	0.1	0.6	3	0.4	BDL	BDL	BDL	BDL
140	BDL	84	BDL	306	158	BDL	230	91	BDL	BDL	3.2	9.0	0.1	1.2	1.6	0.4	0.6	BDL	BDL	BDL
141	BDL	78	BDL	243	134	BDL	375	111	BDL	BDL	34	6.0	0.1	0.7	BDL	6.2	0.3	BDL	BDL	0.2
142	BDL	133	BDL	408	166	BDL	766	36	BDL	BDL	5.1	28.9	BDL	1.1	1.7	0.7	0.6	BDL	BDL	0.1
143	BDL	191	BDL	465	226	BDL	1066	104	BDL	BDL	2.2	22.9	BDL	1.1	2.4	0.6	0.6	BDL	BDL	BDL
144	BDL	290	BDL	305	148	BDL	596	353	BDL	BDL	2.1	18.7	BDL	0.8	3.4	0.2	BDL	BDL	BDL	BDL
145	BDL	239	BDL	617	231	BDL	403	160	2.3	0.6	8.7	71.7	0.6	1.8	5.7	2	1.5	BDL	BDL	0.1
146	BDL	103	BDL	393	145	BDL	417	51	BDL	BDL	3.7	14.7	BDL	1.0	3.8	0.8	0.4	BDL	BDL	BDL
147	BDL	121	BDL	438	135	BDL	402	93	BDL	1.0	4.9	43.2	BDL	1.0	1.2	0.5	1.4	BDL	BDL	0.1
148	BDL	223	BDL	373	130	BDL	399	344	1.5	BDL	5.4	208	BDL	1.0	2.4	0.6	1.1	BDL	BDL	0.2
149	BDL	135	BDL	421	179	BDL	375	85	BDL	0.4	3.9	16.8	BDL	1.5	3.2	0.3	0.9	BDL	BDL	BDL
150	BDL	156	BDL	291	148	BDL	1051	199	BDL	BDL	2.5	27.6	0.6	1.0	3.5	3.7	0.6	BDL	BDL	BDL
151	702	140	BDL	305	263	BDL	2292	93	1.2	BDL	2.7	83.7	BDL	1.0	3.2	1.3	1.1	BDL	BDL	BDL
152	BDL	173	BDL	416	207	28	971	149	2.6	BDL	3.5	38.9	BDL	1.3	4.7	1.5	0.7	BDL	BDL	BDL
153	BDL	145	BDL	253	87	BDL	746	230	BDL	BDL	2.0	16.2	BDL	0.7	1.4	2	1.4	BDL	BDL	BDL
154	BDL	181	41	289	79	BDL	1516	84	BDL	BDL	39.3	7.4	BDL	1.8	6.3	2.3	0.3	BDL	BDL	0.1
155	BDL	207	BDL	344	93	453	1941	141	BDL	BDL	47.2	5.2	0.1	1.0	4.1	1.9	1.2	BDL	BDL	0.1

BDL = Below detection limit

Table S2. Mineral elements concentration in cultivar familywise (mg/100g).

Family	Na	Mg	Al	P	S	Cl	K	Ca	Ti	Cr	Mn	Fe	Co	Cu	Zn	Rb	Sr	Mo	Ba	Pb
Amaryllidaceae	0	328±8	0	532±15	579±16	0	890±19	520±11	1.7±0.0	0	5.3±0,2	32±1	0	1.0±0.0	5.2±0.1	0.7±0.0	0.9±0.0	0	0	0.1±0.0
Anacardiaceae	0	43±1	0	251±7	120±3	96±3	674±14	98±2	0.2±0.0	0.1±0.0	2.5±0.1	10±0	0	2.4±0.0	3.2±0.1	2.1±0.1	0.5±0.0	0	0	0.1±0.0
Annonaceae	0	46±1	0	120±3	294±8	73±2	557±12	54±1	0	0.4±0.0	3.7±0.1	233±5	0	1.7±0.0	0	1.1±0.0	0.1±0.0	0	0	0.3±0.0
Apiaceae	281±6	255±6	52±1	249±7	279±8	512±15	1934±41	1253±28	6.3±0.2	0.1±0.0	8.6±0.3	81±2	0.1±0.0	1.3±0.0	4.8±0.1	2.6±0.1	13±0	0	2.4±0.1	0.1±0.0
Arecaceae	580±13	76±2	0	125±4	384±10	61±2	510±11	93±2	0.9±0.0	0.6±0.0	5.6±0.0	345±7	0	1.0±0.0	1.4±0.0	1.0±0.0	0	0	0	0.4±0.0
Asparagaceae	0	117±3	0	215±6	216±6	0	388±8	142±3	0	0.5±0.0	1.5±0.0	15±0	0	1.0±0.0	1.7±0.0	0.6±0.0	0	0	0	0
Asteraceae	0	190±5	0	547±16	193±5	4±0	820±17	494±11	0.6±0.0	0	3.8±0.1	58±1	0	1.5±0.0	5.0±0.1	1.1±0.0	1.3±0.0	0	0.5±0.0	0
Basellaceae	0	262±7	0	539±16	269±7	176±5	1431±30	19±0	2.5±0.1	0	5.9±0.2	43±1	0	0.6±0.0	10.3±0.2	0.7±0.0	0.2±0.0	0	0	0.1±0.0
Bixaceae	0	143±4	0	311±9	211±6	0	1371±29	264±6	0	0	5.2±0.2	69±1	0	0.6±0.0	2.1±0.0	2.8±0.1	0.9±0.0	0	0	0.1±0.0
Brassicaceae	0	201±5	2±1	517±15	926±25	0	831±17	497±11	0.5±0.0	0	5.2±0.2	34±1	0	0.5±0.0	5.6±0.1	2.5±0.1	1.8±0.1	0	0.1±0.0	0
Burseraceae	2065±47	910±23	67±1	95±3	73±2	253±8	1022±21	584±13	8.7±0,2	1.6±0.0	8.4±0.3	1100±23	0	0.5±0.0	20.7±0.5	1.1±0.0	2.8±0.1	0.3±0.0	0	0.3±0.0
Caricaceae	0	374±9	0	589±17	782±21	134±4	1927±40	929±20	2.7±0.1	0	9.3±0.3	38±1	0	1.0±0,0	7.5±0.2	3.5±0.1	2.9±0.1	0.2±0.0	0	0.2±0.0
Celastraceae	0	38±1	0	149±4	82±2	0	530±11	372±8	1.6±0.0	0	3.7±0.1	27±1	0	0.7±0.0	1.1±0.0	1.5±0.0	3.2±0.1	0	1.2±0.0	0.1±0.0
Cornaceae	0	95±2	0	303±9	172±5	0	1539±32	40±1	0	0	1.1±0.0	22±0	0	1.2±0.0	2.4±0.1	1.4±0.0	0	0	0	0
Cucurbitaceae	0	146±4	0	536±16	204±6	0	476±10	72±2	0	0.1±0.0	4.6±0.1	19±0	0	1.6±0.0	6.2±0.1	1.4±0.0	0.2±0.0	0	0	0
Dipterocarpaceae	0	50±1	0	138±4	91±2	0	649±14	80±2	0	0	5.5±0.2	2.7±0.1	0.1±0.0	0.8±0.0	1.0±0.0	0.7±0.0	0.5±0.0	0	0	0
Ebenaceae	0	49±1	0	53±2	50±1	0	205±4	508±11	0	0	1.1±0.0	80±2	0	0.6±0.0	0.9±0.0	0.5±0.0	2.2±0.1	0	1.6±0.0	0.1±0.0
Euphorbiaceae	416±10	219±5	0	568±16	212±6	0	681±14	286±6	0	0	1.3±0.0	8.9±1.9	0	1.0±0.0	6.1±0.1	0.9±0.0	0.1±0.0	0	0	0
Fabaceae	79±2	258±6	3±0	473±14	638±17	11±0	1202±25	478±11	0.1±0.0	0.2±0.0	4.6±0.1	18±1	0.1±0.0	1.5±0.0	4.5±0.1	1.6±0.0	1.4±0.0	0.2±0.0	0.1±0.0	0.1±0.0
Juglandaceae	0	0	0	233±7	71±2	0	349±7	64±1	0.9±0.0	0	1.0±0.0	4.5±0.1	0	1.0±0.0	2.1±0.0	0.4±0.0	0.3±0.0	0	0	0
Lauraceae	0	121±3	0	373±11	327±9	0	890±19	198±4	0	0	4.6±0.1	6.5±0.1	0.1±0.0	1.3±0.0	4.2±0.1	1.0±0.0	0.5±0.0	0	0	0
Linaceae	0	151±4	0	273±8	154±4	0	622±13	361±8	0	0	3.4±0.1	8.6±0.2	0.1±0.0	0.9±0.0	5.5±0.1	0.6±0.0	0.3±0.0	0	0	0
Loganiaceae	4305±99	164±4	0	368±11	252±7	0	784±16	262±6	0	0	1.6±0.0	4.7±0.1	0.1±0.0	0.8±0.0	3.6±0.1	2.3±0.1	0.3±0.0	0	0	0
Lecythidaceae	0	210±5	0	437±13	536±14	0	703±15	137±3	0	0.5±0.0	1.7±0.1	14±0	0.1±0.0	4.2±0.0	2.7±0.1	1.3±0.0	0.3±0.0	0	0	0.2±0.0
Lythraceae	26±1	122±3	90±2	251±7	159±4	379±11	1391±29	296±7	1.8±0.1	0.4±0.0	4.2±0.1	74±2	0.1±0.0	2.0±0.0	1.6±0.0	2.2±0.1	1.3±0.0	0	0	0.1±0.0
Malvaceae	312±7	307±8	0	661±19	280±8	0	1156±24	536±12	0.2±0.0	0	5.3±0.2	17±0	0.1±0.0	2.3±0.0	5.6±0.1	1.3±0.0	1.1±0.0	0	0.2±0.0	0
Meliaceae	0	131±3	0	385±11	453±12	0	1107±23	175±4	0.8±0.0	0	1.6±0.0	16±0	0	1.0±0.0	5.5±0.1	1.6±0.0	0.4±0.0	0	0	0
Moraceae	0	233±6	0	293±8	159±4	83±2	2398±50	603±13	2.6±0.1	0.4±0.0	3.8±0.1	35±1	0.1±0.0	0.9±0.0	2.0±0.0	3.0±0.1	1.8±0.1	0.1±0.0	0	0.1±0.0
Myrtaceae	509±12	72±2	0	189±5	130±4	0	553±12	37±1	0	0	0.7±0.0	21±0	0.1±0.0	0.9±0.0	0.9±0.0	1.1±0.0	0	0	0	0
Moringaceae	0	317±8	0	602±17	2067±56	0	1006±21	118±3	0	0	2.3±0.1	8.4±0.2	0.1±0.0	0.6±0.0	4.3±0.1	0.5±0.0	0.1±0.0	0	0	0
Nelumbonaceae	0	176±4	0	571±17	284±8	0	1310±28	201±4	0	0.3±0.0	7.9±0.2	19±0	0	2.0±0.0	3.5±0.1	3.6±0.1	1.7±0.0	0	0	0.1±0.0
Papeveraceae	0	71±2	0	341±10	141±4	0	482±10	825±18	0	0	5.4.0.2	15±0	0.2±0.0	1.9±0.0	4.5±0.1	0.9±0.0	4.8±0.1	0	0.7±0.0	0
Pedaliaceae	0	119±3	0	323±9	178±5	0	328±7	919±20	1.3±0.0	0	2.2±0.1	25±0	0.1±0.0	0.8±0.0	3.5±0.1	0.6±0.0	3.6±0.1	0	2.3±0.1	0
Piperacee	476±17	143±4	0	176±5	121±3	373±11	1924±40	467±10	0	0	6.1±0.2	10±0	0.1±0.0	1.8±0.0	0	4.6±0.1	2.3±0.1	0	0	0.2±0.0
Polygonaceae	448±13	204±5	0	422±12	85±2	0	809±17	80±2	0	0	7.8±0.2	81±2	0	0.5±0.0	2.8±0.1	1.2±0.0	0.4±0.0	0.1±0.0	0	0
Passifloraceae	0	45±1	0	321±9	145±4	0	565±12	39±1	0	0	1.1±0.0	11±0	0	1.0±0.0	3.4±0.1	0.5±0.0	0	0	0	0
Putranjivaceae	1290±37	242±6	0	259±8	1587±43	0	1324±28	1059±23	0	0	0.9±0.0	5.3±0.1	0.1±0.0	1.1±0.0	1.0±0.0	1.5±0.0	3.0±0.1	0	0	0
Phyllanthaceae	0	63±2	0	204±6	98±3	0	1498±31	343±8	0	0	1.3±0.0	15±0	0	1.0±0.0	1.4±0.0	1.1±0.0	0.7±0.0	0	0	0
Ranunculaceae	0	115±3	0	404±12	227±6	0	653±14	745±16	0	0	3.4±0.1	9.0±0.2	0.1±0.0	1.0±0.0	5.7±0.1	1.2±0.0	3.6±0.0	0	1.2±0.0	0
Rhamnaceae	0	149±4	0	461±13	337±9	0	707±15	146±3	0	0	4.1±0.1	15±0.3	0.1±0.0	1.0±0.0	11.7±0.3	1.6±0.0	0.3±0.0	0	0	0
Rosaceae	0	94±2	0	306±9	107±3	0	596±13	199±4	0	0	1.8±0.1	4.9±0.1	0.1±0.0	0.9±0.0	3.2±0.1	0.6±0.0	1.0±0.0	0	0	0
Rubiaceae	283±7	71±2	0	76±2	68±2	0	1480±31	356±8	0.7±0.0	0	1.8±0.1	22±0	0	0.9±0.0	0.7±0.0	1.1±0.0	1.0±0.0	0.1±0.0	0	0.1±0.0
Rutaceae	0	70±2	0	255±7	127±3	0	869±18	488±11	0.8±0.0	0.2±0.0	3.5±0.1	39±1	0.1 ±0.0	0.7±0.0	1.9±0.0	0.7±0.0	2.5±0.1	0	0.3±0.0	0
Santalaceae	6419±148	122±3	0	319±9	218±6	55±2	589±12	164±4	0	0	0.5±0.0	5.0±0.1	0	0.7±0.0	2.6±0.1	0.9±0.0	0.3±0.0	0	0	0
Sapindaceae	0	41±1	0	275±8	118±3	0	595±12	109±2	0	0	1.5±0.0	7.2±0.2	0.1±0.0	0.9±0.0	1.8±0.0	0.5±0.0	0.3±0.0	0	0	0
Schisandraceae	0	78±2	0	243±7	134±4	0	375±8	111±2	0	0	34±1	6.0±0.1	0.1±0.0	0.7±0.0	0	6.2±0.2	0.3±0.0	0	0	0.2±0.0
Solanaceae	64±2	173±4	0	403±12	180±5	6±0	794±17	152±3	0.8±0.0	0.2±0.0	4.1±0.1	52±1	0.1±0.0	1.1±0.0	3.2±0.1	1.1±0.0	0.8±0.0	0	0	0
Verbenaceae	0	145±4	0	253 ±7	87±2	0	746±16	230±5	0	0	2.0±0.1	16±0	0	0.7±0.0	1.4±0.0	2.0±0.1	1.4±0.0	0	0	0
Zingiberaceae	0	194±5	21±1	317±9	86±2	227±7	1729±36	113±2	0	0	43±1	6.3±0.1	0.1±0.0	1.4±0.0	5.2±0.1	21±0.1	0.8±0.0	0	0	0.1±0.0
